# When AI Is—and Is Not—Needed: AI Literacy and Computational Thinking in Primary STEM

**DOI:** 10.3390/jintelligence14100257

**Published:** 2026-10-12

**Authors:** Ahmet Akif Erbaş, Tolga Topçubaşı

**Affiliations:** 1 Ahmet Erdoğan School of Health Services, Zonguldak Bülent Ecevit University, 67100 Zonguldak, Türkiye; 2 Faculty of Education, İstanbul 29 Mayıs University, 34764 İstanbul, Türkiye

**Keywords:** artificial intelligence literacy, critical AI literacy, computational thinking, integrated STEM education, educational robotics, primary education, machine learning, human-centered AI

## Abstract

Artificial intelligence (AI) is increasingly present in children’s lives, yet exposure does not necessarily lead to an accurate understanding of how AI works. This study examined whether a LEGO-based integrated STEM intervention could improve third-grade students’ AI literacy and computational thinking, particularly their ability to distinguish machine learning from rule-based automation and reason about training data, model error, and technology choice. An embedded mixed-methods, quasi-experimental pretest–posttest control-group design was conducted with 93 students from four intact classrooms. The experimental group (*n* = 46) completed eight weeks of authentic design problems, while the control group (*n* = 47) continued regular Life Studies instruction. After controlling for pretest scores, the experimental group significantly outperformed the control group in AI literacy, F(1, 90) = 152.51, *p* < .001, partial η^2^ = 0.629, and computational thinking, F(1, 90) = 22.18, *p* < .001, partial η^2^ = 0.198. Qualitative evidence showed a shift from viewing AI through robots and automatic actions toward reasoning about learning from examples, data diversity, limitations, human oversight, accessibility, and problem–technology fit. Integrated STEM instruction can foster critical AI literacy by making AI mechanisms visible and inviting students to judge whether AI is needed at all.

## 1. Introduction

Children increasingly encounter AI through recommendation systems, voice assistants, image recognition, and generative applications. These systems may feel familiar because they respond quickly and appear intelligent, but familiarity with an interface does not reveal what data the system uses, how its outputs are produced, or why it may fail. Primary education therefore faces a task that is broader than teaching children to operate new tools. Students need opportunities to examine what makes a system artificially intelligent, how it differs from ordinary automation, and whether AI is an appropriate response to a problem.

This study examines whether an eight-week, LEGO-based integrated STEM intervention can develop third-grade students’ AI literacy and computational thinking as related but distinct outcomes. Its distinctive feature is a sustained contrast between machine learning and rule-based automation within comparable physical systems, so that deciding when AI is—and is not—needed becomes part of what students learn, assessed with performance tasks, a comparison group, and weekly process evidence.

## 2. Theoretical Framework and Literature Review

### 2.1. Theoretical Perspective

Three connected perspectives guided the design and assessment. Constructionism (Papert, 1980; Papert & Harel, 1991) holds that learners build knowledge by constructing public, testable artifacts; students therefore built ideas such as “the system learns from examples” or “the system follows a rule written in advance” into LEGO models whose behavior they could observe and revise, treating unexpected outputs as evidence rather than failure. Integrated STEM is understood as design-based problem solving in which engineering design connects disciplinary ideas to an authentic problem (Kelley & Knowles, 2016); Life Studies supplied the problems, and the design cycle was also the source of the computational thinking practices examined. AI literacy is treated as a multidimensional competence (Long & Magerko, 2020; Ng et al., 2021) extended in a critical, human-centered direction (Stolpe & Hallström, 2024; Yim, 2024) to include what a system actually recognizes and whether AI is needed at all. These perspectives shaped the contrastive design, the eight assessment domains, and the four competency profiles.

### 2.2. AI Literacy as a Multidimensional and Critical Construct

AI literacy has been defined as the knowledge and practices needed to understand, use, evaluate, and communicate about AI while considering its ethical and social implications (Long & Magerko, 2020; Ng et al., 2021). Reviews of K–12 research describe a similarly multidimensional construct that includes conceptual knowledge, data-related reasoning, computational practices, creation, critical evaluation, and ethics (Casal-Otero et al., 2023; Lee & Kwon, 2024; Liu & Zhong, 2024; Rizvi et al., 2023). Work focused on children and primary education further stresses that these dimensions must be translated into developmentally appropriate experiences rather than simplified into tool use or abstract terminology (Atias & Mawasi, 2025; Yim, 2024; Yim & Su, 2025).

This multidimensionality is important because AI literacy can otherwise be reduced either to operational skill or to general awareness. A technically informed account includes knowledge of inputs, models, training, classification, and uncertainty, while a socio-technical account also asks who designed the system, whose data are represented, what forms of human judgment remain necessary, and how consequences are distributed. Reviews spanning K–12 education show that research has often emphasized applications and attitudes more strongly than students’ mechanistic and evaluative understanding (Biagini, 2025; Martin et al., 2024). Stolpe and Hallström (2024) similarly argue that AI literacy in technology education should combine technological-scientific knowledge with socio-ethical understanding. For primary learners, these dimensions need not be taught as separate units. A single design problem can require students to identify the mechanism, test its limits, consider affected users, and defend a technology choice.

The way AI literacy is assessed should reflect this breadth. Recent work on objective AI literacy measures has emphasized that self-reported familiarity and actual conceptual performance are not interchangeable (Chiu et al., 2024). This issue is especially consequential with young children, who may confidently describe a robot as intelligent without being able to explain whether it learned from data or followed a programmed condition. Scenario-based tasks, explanations, and design decisions can therefore provide evidence that is closer to the intended construct than perceived competence alone. The present study adopted this performance orientation and supplemented it with repeated process evidence so that changes in total scores could be interpreted alongside changes in students’ explanations.

Several terms recur in this article. A machine learning (AI) system derives its decision rule from labeled training examples and classifies new input (e.g., a pose classifier assigning a new body pose to a learned class), whereas rule-based (non-learning) automation responds to conditions written in advance by people (e.g., “if the sensor detects movement, turn on the light”) and does not learn. Model error is an incorrect output of a trained model, often reflecting its training examples rather than faulty code; data diversity is how well those examples represent different users and conditions. Human oversight means that a person checks an output and remains responsible when the consequence matters, accessibility concerns whether all intended users can operate a system, and AI necessity is the judgment of whether a problem requires learning or classification or can be solved with a fixed rule.

### 2.3. Children’s Conceptions of AI and Rule-Based Automation

One persistent difficulty is that young learners may identify AI through visible features. Robots, sensors, movement, speech, or automatic responses can all be interpreted as evidence of intelligence. Primary students have been found to associate AI with robots, computers, human-like machines, and independent action (Walan, 2025). Such ideas are understandable because the internal operation of AI is hidden while the output is observable. They nevertheless blur an important distinction: a device may follow an explicit rule written in advance, or it may classify new input by using patterns learned from examples. A sensor-controlled light and a pose classifier can both respond automatically, but they do not make decisions in the same way.

Learning this difference is more than a matter of terminology. If every automatic system is labeled AI, students may overestimate what AI is and overlook the role of human-authored rules. By comparing mechanisms, they can ask more precise questions: Did the system learn from examples? Does it always follow the same condition? What information does it actually process? Would a different user or setting change the output? Contrastive examples can therefore move attention away from a device’s appearance and toward the process that produces its behavior.

The comparison also opens a question that receives less emphasis in many AI education activities: Is AI needed? Current frameworks understandably emphasize understanding, applying, creating, and evaluating AI. Yet an AI-first orientation can develop when the newest technology is treated as the preferred solution regardless of the problem. Many everyday tasks can be addressed more transparently through a timer, sensor, button, or fixed if–then rule. Critical AI literacy should include the ability to compare alternatives and justify why a problem does—or does not—require learning, classification, or prediction.

### 2.4. Data, Error, Oversight, and Accessibility

Judging AI also depends on understanding data. A machine learning model does not comprehend the social meaning attached to its output. Its performance is shaped by the input it receives, the labels and categories defined by people, the examples included during training, and the conditions encountered during use. Data-related concepts are therefore central to K–12 AI education (Olari & Romeike, 2024). They are also closely connected with wider calls for students to reason with data in meaningful STEM contexts rather than encounter data literacy as an isolated topic (Friedrich et al., 2024). For young learners, comparing many similar examples with fewer but more varied examples can provide an accessible introduction to representation and generalization.

Model error is especially useful for making these relationships visible. A classifier trained with one child at one distance may work well during training but fail when another child performs the same movement. If students treat this only as a malfunction, they miss the connection between the training set and system behavior. If they test different users, identify patterns in failure, revise the examples, and compare the results, error becomes evidence. This process can also support early reasoning about human oversight. Once students observe that a system can be wrong, they can consider when an output should be checked and who remains responsible for an important decision. Reviews of responsible AI education argue that ethical awareness should be accompanied by opportunities to consider responses and mitigation, not merely identify a problem (Ma et al., 2025), and intervention research with older students shows that such reasoning can be taught (Usher & Barak, 2024).

Accessibility provides another concrete route into human-centered AI literacy. A gesture-controlled system may function technically while excluding users who cannot perform the expected movement. Asking who can use the system, who may be left out, and which alternative inputs are possible connects technical design with inclusion. These questions do not require primary students to master advanced ethical vocabulary. They require them to examine a design from the standpoint of different users and revise it accordingly.

### 2.5. Learning About AI Through Integrated STEM and Educational Robotics

Such concepts are difficult to teach through explanation alone. Constructivist, collaborative, project-based, and design-oriented approaches can make AI more accessible because learners build, test, and discuss systems rather than receive finished descriptions (Casal-Otero et al., 2023; Liu & Zhong, 2024; Shamir & Levin, 2022). Elementary studies combining AI with STEAM and problem-based learning also suggest that authentic tasks can connect conceptual learning with interdisciplinary design (Cheng et al., 2025). However, the evidence base still includes many short interventions, small samples, self-report measures, and studies without comparison groups (Liu & Zhong, 2024; Rizvi et al., 2023).

Age-appropriate AI education is therefore a problem of pedagogical representation as much as content selection. Yang (2022) proposes that early AI curricula should make a limited set of core ideas learnable through meaningful experience rather than reproduce advanced technical courses in simplified language. Tangible and embodied activities are useful in this respect because the child can supply examples, observe the response, change one condition, and test again. Shamir and Levin (2022) showed that elementary learners can engage with machine learning concepts when the learning environment supports experimentation and explanation. The present intervention followed the same principle but added a sustained contrast with non-learning automation, allowing students to examine both the presence and absence of machine learning within comparable physical systems.

Integrated STEM offers a promising setting because it organizes learning around problems that require knowledge and practices from more than one field. AI can be connected with science and STEM through modeling, data, evidence, programming, and design (Park et al., 2023). Educational robotics can further make sequences, conditions, inputs, outputs, and errors observable. Its effects, however, depend on pedagogy. A meta-analysis found positive effects of educational robotics on several STEM outcomes but only a small, nonsignificant average effect on computational thinking, indicating that access to robotics alone is insufficient (Ouyang & Xu, 2024). Carefully structured cycles of problem definition, algorithm design, testing, debugging, and redesign are more likely to support learning (Chevalier et al., 2020).

The intersection of AI and STEM can take two different forms. AI may be used as a tool to support STEM learning, or AI itself may become content that students investigate through STEM practices. Xu and Ouyang’s (2022) review of AI applications in STEM education illustrates the diversity of the first form, whereas the present study is closer to the second: students treated AI systems as designed, testable, and revisable technological models. This orientation is important because merely embedding an AI-enabled product in a STEM activity does not ensure AI literacy. Students need to reason about the data and decision mechanism inside the solution as well as the disciplinary problem outside it.

Research on educational robotics also suggests that the quality of scaffolding and the organization of interaction shape learning. Primary and early-years studies report gains when learners actively program, debug, predict, and explain robot behavior (Angeli & Valanides, 2020; Chalmers, 2018; Noh & Lee, 2020; Sung et al., 2023). Reviews of robotics with young learners likewise emphasize that construction materials become educationally productive when tasks direct attention to computational ideas and iterative problem solving rather than only to assembly or motivation (Bakala et al., 2021; Ching & Hsu, 2024). These findings informed the recurring structure of the intervention and the decision to rotate roles so that coding, data, design, and testing responsibilities were not concentrated in the same students each week.

### 2.6. Computational Thinking and AI Literacy

Computational thinking and AI literacy overlap in these cycles but are not interchangeable. Building a classifier may require decomposition, sequencing, conditional reasoning, testing, and revision. Understanding why training examples matter, what the model actually recognizes, who is represented, and whether AI is appropriate requires additional conceptual and evaluative reasoning. For example, in the pedestrian-crossing task, ordering the blocks that switch the signal and linking a predicted class to an output are computational thinking practices, whereas explaining why the classifier recognizes one child but not another, and whether a push button would serve better, requires AI literacy; the two overlap in testing and debugging but differ in whether the algorithm or the data–model–user relationship is being reasoned about. Research on computational thinking in primary education shows that young learners can develop computational thinking skills, such as algorithmic thinking and debugging, through structured curricula and the programming of tangible robots (Falloon, 2024; Misirli & Komis, 2023), while reviews of CT–STEM integration report transfer that varies by design, duration, and learner characteristics (Li & Oon, 2024; Montuori et al., 2024). The relationship between AI literacy and computational thinking should therefore be examined empirically rather than assumed.

### 2.7. The Present Study

The present study addressed these needs through an eight-week LEGO-based integrated STEM intervention with third-grade students. Life Studies problems involving school safety, movement, recycling, energy, pedestrian safety, accessibility, and community needs provided the context. Students designed both machine learning and rule-based systems, tested their operation, investigated errors, and justified whether AI was necessary. The study contributes to the Special Issue’s focus on the intersection of AI literacy and STEM education by examining AI literacy and computational thinking as related but distinct outcomes, using a comparison group and combining performance measures with process evidence.

The study addressed four research questions:

1. What effect does LEGO-based integrated STEM instruction have on third-grade students’ AI literacy after initial performance is controlled?
2. What effect does the intervention have on students’ computational thinking after initial performance is controlled?
3. How do students’ AI competency profiles and explanations of AI, automation, data, error, oversight, accessibility, and AI necessity change during the intervention?
4. How do students’ design, testing, debugging, and redesign practices help explain the observed development?

## 3. Materials and Methods

### 3.1. Research Design

The study used an embedded mixed-methods, quasi-experimental pretest–posttest control-group design. Four intact third-grade classrooms participated: two formed the experimental group and two the control group. Students remained in their existing classes, so individual random assignment was not possible. The quantitative strand tested group differences in AI literacy and computational thinking. The embedded qualitative and process strand examined how students explained system mechanisms and how their problem solving developed during the same eight-week period.

The two outcomes were specified separately. Computational thinking referred to performance involving sequences, loops, conditions, algorithmic reasoning, testing, and problem solving. AI literacy referred to the ability to distinguish learned classification from rule-based automation, explain training examples, identify the system’s actual input, interpret error, evaluate data diversity, consider oversight and accessibility, and judge whether AI matched the problem.

### 3.2. Participants and Context

Participants were 93 third-grade students enrolled in four classrooms at a primary school in Türkiye. The experimental group included 46 students (24 and 22 in the two classrooms), and the control group included 47 students (23 and 24). Convenience sampling was used because the intervention required continuing access to a programmable construction and AI environment, suitable infrastructure, regular attendance, and permission to document classroom processes. The study was conducted in the spring semester of the 2025–2026 school year.

All students completed the computational thinking and AI literacy assessments before and after the intervention. Experimental-group students worked in teams of three or four (ten teams of four and two of three), each team sharing one kit, with four rotating responsibilities: design and modeling (building and adapting the LEGO model), coding and algorithm (planning and programming the sequence of operations), data and AI (collecting and labeling pose examples and training the classifier in AI weeks, and defining the sensor or event conditions in rule-based weeks), and testing and improvement (testing the system with different users and conditions, recording errors, and proposing improvements). The roles changed weekly to distribute participation across the design cycle so that each student held each role twice. The two teams of three rotated through the same four roles: each week one role was left unassigned on a rotating basis (each role in two of the eight weeks) and its tasks were shared by the three members, so that these students also held each role twice. Individual tests, micro-tasks, and interview responses were analyzed as individual evidence; worksheets, code, models, and products were treated as group-level evidence.

### 3.3. Instructional Design

The experimental group participated in an eight-week integrated STEM sequence supported by a programmable construction and AI kit (LEGO Education Computer Science & AI). Life Studies provided age-appropriate problems connected with school, health, safety, the environment, accessibility, and community life. Every activity followed the same broad cycle: define the problem and affected users; decompose the task; choose an AI or rule-based approach; plan and construct the system; program rules or train a classifier; test; identify errors; revise; and justify whether the final technology was appropriate.

The contrast between machine learning and automation was deliberate. Pose-recognition activities were treated as AI because students trained a classifier with labeled examples and tested its classification of new input. Systems controlled by events, sensors, loops, variables, or predefined if–then conditions were treated as rule-based automation. Students repeatedly explained how each system made decisions rather than labeling it from its appearance.

The weekly sequence was cumulative (Table 1). Weeks 1 and 4 established rule-based reference points through safety and energy systems in which events or sensor values triggered predetermined responses. Weeks 2 and 3 introduced pose classification by asking students to collect labeled movement examples and connect predicted categories to LEGO outputs. The recycling task deliberately separated technical input from social meaning: the classifier did not inspect paper, plastic, or glass, but recognized a pose to which students had assigned one of those meanings. This distinction became a recurring question in later activities.

Weeks 5–7 shifted from basic operation to generalization and responsibility. Students tested pedestrian and assistance systems with different classmates, distances, angles, lighting conditions, and movement styles. When the classifier failed, they compared code errors with data-related errors and retrained it using more varied examples. Accessibility was introduced by asking whether all intended users could perform the required gesture and by considering sound, buttons, or simpler movements as alternatives. In Week 8, groups selected a community problem and justified an AI or automation solution. Their final explanation had to identify users, input, decision mechanism, possible failure, need for oversight, accessibility, and whether AI added value beyond a fixed rule.

**Figure 1:**
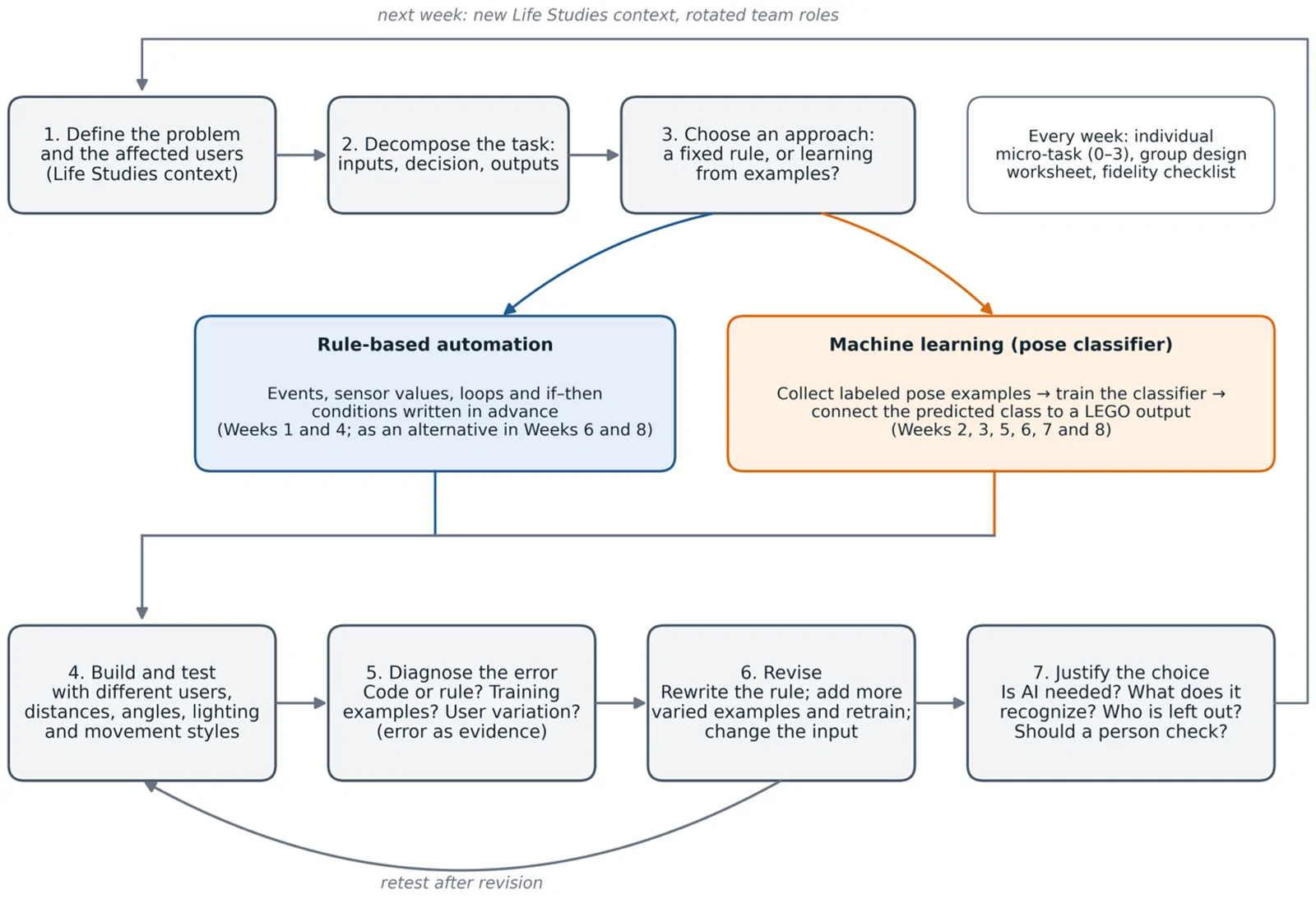
The weekly design cycle. After decomposing the problem, teams followed a rule-based or a machine learning (pose classifier) pathway; both converged on testing with different users, error diagnosis, revision, and a justification of whether AI was needed. Blue and orange boxes and arrows indicate the rule-based and machine learning pathways, respectively; gray boxes and arrows show the steps shared by both. The line along the top leads to the next week’s problem, and the curved arrow at the bottom indicates retesting after revision.

**Figure 2:**
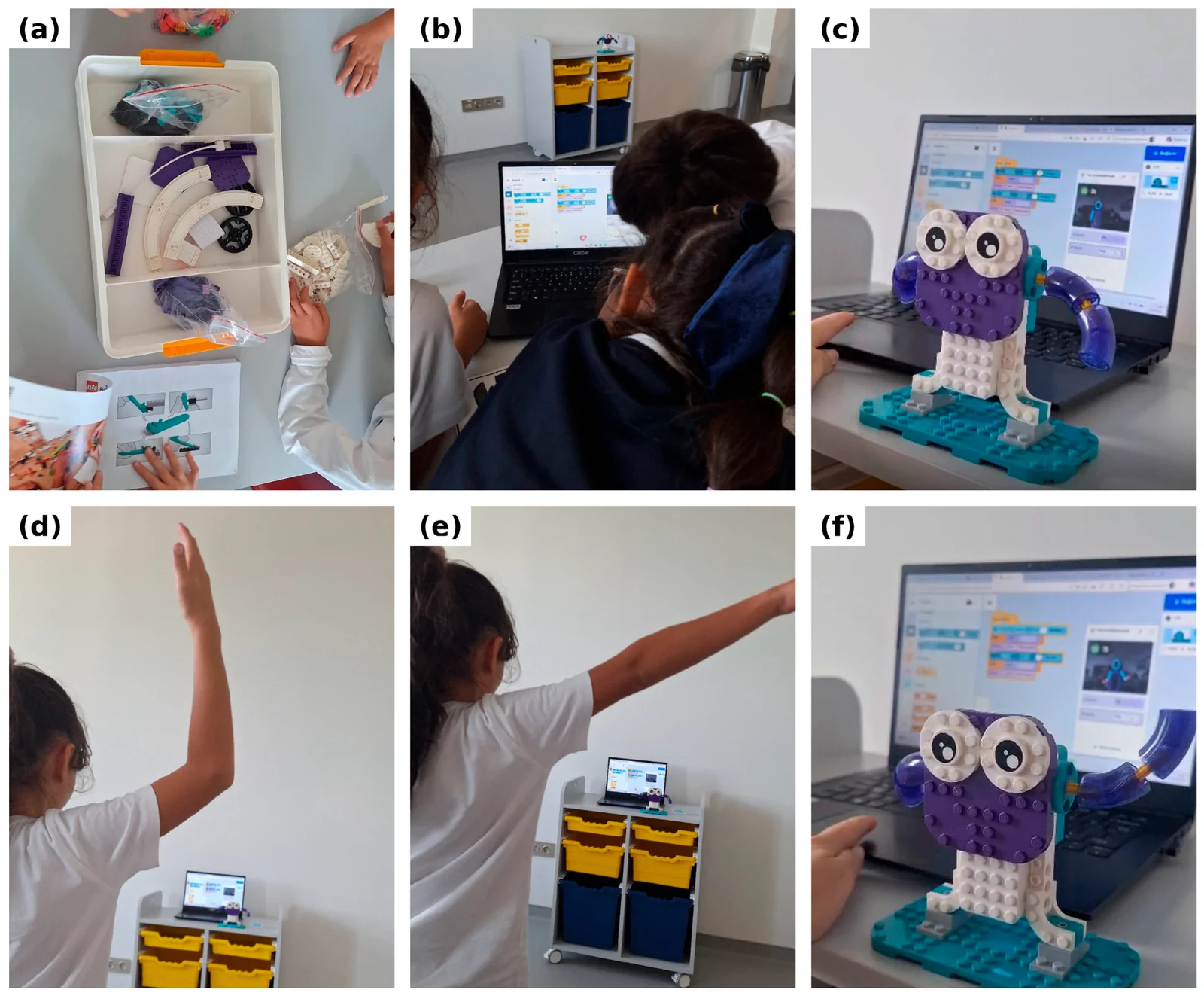
Classroom implementation: (**a**) a team building a model from the kit; (**b**) programming the model in the block-based environment; (**c**) the model beside the program and the pose-classification panel; (**d**,**e**) students performing two poses for the camera during training and testing; (**f**) the model’s motorized arm moving during testing. No faces are shown; images were taken with parental consent.

Each weekly activity was delivered in two sessions (about 105–135 min in total) according to a written implementation guide (Appendix A; File S2) and followed the cycle in Figure 1. Teams discussed a real-life Life Studies problem and narrowed it to a specific case (e.g., in Week 1, each team chose one school-safety problem), decided whether it required a fixed rule or learning from examples, built the model, and programmed the rule or trained the pose classifier. They then tested the system with different students and conditions, located errors, revised the system, completed a team form, and answered an individual micro-task. Table 1 lists each week’s task, Figure 2 shows the classroom work, and Appendix A gives the plans for Weeks 1 and 7.

### 3.4. Instructors, Facilitation, and Implementation Fidelity

All experimental sessions were delivered by the second author, who has taught educational robotics and AI for more than 10 years, holds certification in robotics coding and AI education, was trained on the LEGO Education Computer Science & AI kit, and used LEGO robotics sets extensively as a classroom teacher. The classroom teachers observed and supported but did not teach, and no teacher professional development was included, so the findings reflect learning under expert facilitation (Section 7).

Instruction was facilitative rather than answer-giving. Students were asked what problem they were solving, what information the system used, whether it learned from examples or followed a rule, why it failed, whether it would work for another person, who might be unable to use it, whether a person should check the output, and whether AI was necessary. The instructor did not immediately identify a system as AI or tell students to add diverse examples when a classifier failed. The implementation guide specified the neutral prompts to be used and the statements to be avoided (e.g., “This system is not AI”; Appendix A).

A weekly fidelity checklist recorded consistent presentation of the problem context, rotation of roles, student-generated solutions, algorithm development, testing, discussion of error, opportunities for redesign, and independent completion of micro-tasks. AI weeks were checked for genuine use of training and classification; automation weeks were checked to ensure that fixed-rule systems were not mislabeled as AI.

### 3.5. Control Condition

The two control classrooms continued regular Life Studies lessons during the same eight weeks, taught by their own teachers with the national curriculum and textbook. They covered the same themes (school and traffic safety, health, recycling, energy saving, and community life) through reading, teacher explanation, discussion, worksheets, and drawing or poster work, without construction kits, coding, or AI tools and without any instruction on AI, data, or computation. This business-as-usual comparison controls for maturation, retesting, and the shared content but is not an active comparison (Section 4.4 and Section 7).

### 3.6. Data Collection

#### 3.6.1. Computational Thinking Skills Test

Computational thinking was measured with the Turkish adaptation of the Competent Computational Thinking Test. Its 23 visual multiple-choice items assess sequences, simple and complex loops, conditional and while structures, and combinations of computational concepts. Correct responses are scored 1 and incorrect or unanswered responses 0, yielding a total from 0 to 23. The Turkish adaptation reported KR-20 = 0.78 (Sever et al., 2025). Students completed the test individually at pretest and posttest.

#### 3.6.2. AI Literacy Performance Assessment

Because no available standardized measure matched both the age group and the intervention’s conceptual aims, an eight-task performance assessment was developed. The open-ended tasks addressed: (a) AI versus rule-based automation, (b) training data and classification, (c) actual system input, (d) data diversity and different users, (e) error and human oversight, (f) accessibility and human-centered AI, (g) data quantity versus diversity, and (h) whether AI was necessary.

Form A was used at pretest and Form B at posttest; both contain the same eight tasks in the same order and use the same rubric, but each task is set in a different everyday scenario to reduce memory effects (e.g., a motion-sensor corridor light in Form A and an irrigation system that starts at 08:00 in Form B). Both forms and the rubric are provided in Appendix B. Each response was scored from 0 to 3: 0 indicated missing, irrelevant, or incorrect reasoning; 1 a superficial judgment based mainly on visible technology or automatic behavior; 2 a broadly accurate but incomplete explanation; and 3 an accurate, justified explanation referring to the relevant mechanism, data issue, limitation, user need, or technology-selection principle. Total scores ranged from 0 to 24. Specialists in primary education, educational technology or AI, measurement, and classroom teaching reviewed the content, wording, developmental appropriateness, and scoring criteria. Cognitive interviews and pilot applications with third-grade students were used to examine comprehensibility and the parallel forms. Responses were scored with the rubric after joint calibration on an initial set of responses, and uncertain cases were resolved by consensus; formal inter-rater agreement coefficients were not computed (Section 7).

The assessment was intended to capture increasingly sophisticated forms of reasoning rather than isolated vocabulary. For example, a student who stated that a device was AI because it moved received a lower score than a student who referred to learning from examples; a student who simply recommended adding more data received less credit than one who explained why examples from different users or conditions were needed. The same principle applied to oversight, accessibility, and AI necessity. The scoring rubric therefore linked conceptual accuracy with justification. This performance orientation is consistent with recent calls to complement AI literacy self-reports with objective tasks that reveal what students can recognize, explain, and evaluate (Chiu et al., 2024).

The four competency profiles offered an additional interpretive layer. Technology-centered responses relied mainly on visible features such as robots, sensors, or automatic action. Functional understanding recognized that an AI system performs a task but provided limited explanation of data or learning. Data- and learning-based understanding referred to examples, training, classification, input, or model limitations. Critical and human-centered understanding combined these mechanisms with reasoning about oversight, inclusion, consequences, or whether AI was appropriate. Profiles were assigned from patterns across tasks rather than a single cut score, and they were used descriptively rather than as a validated universal developmental sequence.

#### 3.6.3. Process and Qualitative Evidence

At the end of each week, experimental-group students completed an individual micro-task scored from 0 to 3. The tasks addressed different concepts and were not treated as equivalent repeated measures; their distributions were used as process indicators. Groups also completed design worksheets documenting the problem, affected users, inputs or data, algorithm, initial test, error, and redesign. Photographs, coding records, test notes, and final products were collected. The final products were examined with a 12-dimension rubric covering problem definition, Life Studies connection, decomposition, algorithmic structure, technology choice, pose recognition where relevant, training data, testing and debugging, redesign, user needs, limitations, and AI necessity.

Post-intervention focus-group interviews involved 12 students selected to represent high, moderate, and limited development in AI literacy. The eight questions (Table A5) concerned AI and automation, learning from examples, error, data diversity, oversight, accessibility, technology choice, and change in thinking—for example, “What is the most important difference between AI and a system that works automatically?”, “Which is more useful: many identical examples or fewer but different examples? Why?”, and “Is it necessary to use AI for every real-life problem?” Researcher observations documented decomposition, algorithm design, training decisions, testing, debugging, user considerations, and changes in students’ explanations. Interview statements were analyzed as individual evidence, whereas collaborative artifacts remained group-level evidence.

### 3.7. Data Analysis and Integration

The quantitative data were screened for missing values, entry errors, implausible values, outliers, and distributional problems. Descriptive statistics, Shapiro–Wilk tests, histograms, Q–Q plots, skewness, and kurtosis were considered together. Independent-samples *t* tests examined pretest differences. Paired-samples *t* tests examined within-group change. Separate ANCOVAs were then conducted for AI literacy and computational thinking, with posttest score as the dependent variable, group as the independent variable, and the corresponding pretest as covariate. Linearity, homogeneity of regression slopes, and homogeneity of error variance were checked. Adjusted means and partial eta squared were reported, with α = 0.05.

The eight AI literacy domains were analyzed separately: pre–post change was tested within each group with paired *t* tests (Cohen’s d for paired data) and between-group differences in gains with Welch’s *t* tests, applying a Bonferroni-adjusted α of 0.006 to each family of eight tests; Wilcoxon and Mann–Whitney tests served as nonparametric checks. Gain-score correlations explored the association between AI literacy and computational thinking across the full sample and within each condition. Students were also classified into four competency profiles—technology-centered, functional, data- and learning-based, and critical and human-centered—using total performance together with response patterns on the core conceptual indicators. Profile transitions were examined from pretest to posttest.

Weekly explanations, interviews, observations, worksheets, and products were analyzed thematically. Deductive categories reflected the research questions, including AI–automation distinction, learning from examples, actual input, data diversity, error, oversight, accessibility, AI necessity, decomposition, algorithmic reasoning, testing, debugging, and redesign. Inductive coding captured patterns not adequately represented by the initial categories. A second researcher independently coded a portion of the material to calibrate the codebook; disagreements were resolved through discussion and documented in an audit trail, and inter-coder agreement coefficients were not computed.

Integration occurred during interpretation. Quantitative outcome changes were compared with domain scores, profile transitions, weekly responses, observations, interviews, and products. Convergence was noted when sources supported the same interpretation; complementarity when one source clarified another; and divergence when evidence did not align. This approach distinguished outcome change from changes in process and explanation.

Trustworthiness was strengthened by maintaining the distinction between individual and collaborative evidence, triangulating recurring claims across sources, independently coding a portion of the qualitative material, and documenting revisions to the coding framework. Quotations were selected to represent repeated patterns or meaningful contrasts rather than to stand alone as proof. Where group products appeared more advanced than some members’ individual explanations, the difference was retained rather than treating the artifact as evidence that every student had reached the same level.

## 4. Results

### 4.1. Preliminary Analyses

The distributions and diagnostic checks supported parametric analysis. The groups did not differ significantly at pretest in AI literacy, t(91) = 1.24, *p* = .218, d = 0.26, or computational thinking, t(91) = −1.24, *p* = .218, d = −0.26. The group-by-pretest interaction was nonsignificant for AI literacy, F(1, 89) = 1.34, *p* = .250, and computational thinking, F(1, 89) = 0.25, *p* = .618, satisfying the homogeneity-of-regression-slopes assumption.

### 4.2. Effects on AI Literacy and Computational Thinking

AI literacy increased markedly in the experimental group and only slightly in the control group (Table 2). After pretest performance was controlled, the adjusted posttest difference was significant, F(1, 90) = 152.51, *p* < .001, partial η^2^ = 0.629. The effect was large and favored students who completed the integrated STEM intervention.

Computational thinking showed the same direction of change, although the effect was smaller. The experimental group increased from M = 10.57 to M = 14.24, whereas the control group changed from M = 11.79 to M = 12.02. ANCOVA showed a significant adjusted posttest difference, F(1, 90) = 22.18, *p* < .001, partial η^2^ = 0.198. Within-group tests (Table 2) showed that the control group’s small increase in AI literacy was also significant, whereas its computational thinking did not change; the ANCOVAs therefore test whether the experimental gains exceeded this baseline change.

### 4.3. Development Across AI Literacy Domains

Experimental-group performance improved significantly in every domain (all *p* < .001, d = 1.17–1.55; Table 3), whereas the control group changed little (d = 0.08–0.48), with only the AI-necessity domain meeting the adjusted criterion. Gains were larger in the experimental group in all eight domains (all *p* < .001, d = 0.87–1.49). The largest absolute change concerned data diversity and different users (1.17 to 2.41), followed by identifying the actual input recognized by the system (1.28 to 2.50). Distinguishing AI from rule-based systems had the highest initial and final means, suggesting that students began with some partial awareness and developed more accurate mechanistic explanations. The lowest pretest means occurred for AI necessity, data quantity versus diversity, and accessibility. These domains improved substantially but remained comparatively demanding at posttest. The total-score increase therefore reflected conceptual, data-related, critical, and human-centered development rather than simple recognition of AI tools.

### 4.4. Changes in Competency Profiles

At pretest, 80.4% of the experimental group fell within the technology-centered or functional profiles, and no student met the criteria for the critical and human-centered profile (Table 4). At posttest, 37.0% were data- and learning-based and 45.7% critical and human-centered. Thus, 82.6% reached the two more advanced profiles. Individual transitions showed that 87.0% moved upward, 13.0% remained stable, and none moved downward. The control group changed less: 25.5% moved upward, 70.2% stayed in the same profile, and 4.3% moved downward.

Because the control classrooms received no instruction on AI, data, or computation (Section 3.5), a between-group difference was expected; the comparison controls for maturation, retesting, and the shared content but does not test the intervention against an alternative pedagogy. The within-group patterns are therefore more informative: which domains proved learnable (Table 3), how many students moved toward data-based and critical reasoning (Table 4), and the form this reasoning took (Section 4.6).

### 4.5. Relationship Between Gains

Across all 93 students, gains in AI literacy and computational thinking were moderately related, r = 0.41, *p* < .001. Within the experimental group, however, the association was smaller and nonsignificant, r = 0.20, *p* = .189; the control-group association was also nonsignificant, r = −0.09, *p* = .531. The full-sample correlation was therefore influenced partly by the experimental group’s greater average gains on both outcomes. Individual development in AI literacy and computational thinking did not proceed in a consistently parallel way within either condition.

### 4.6. Weekly and Qualitative Development

**Figure 3:**
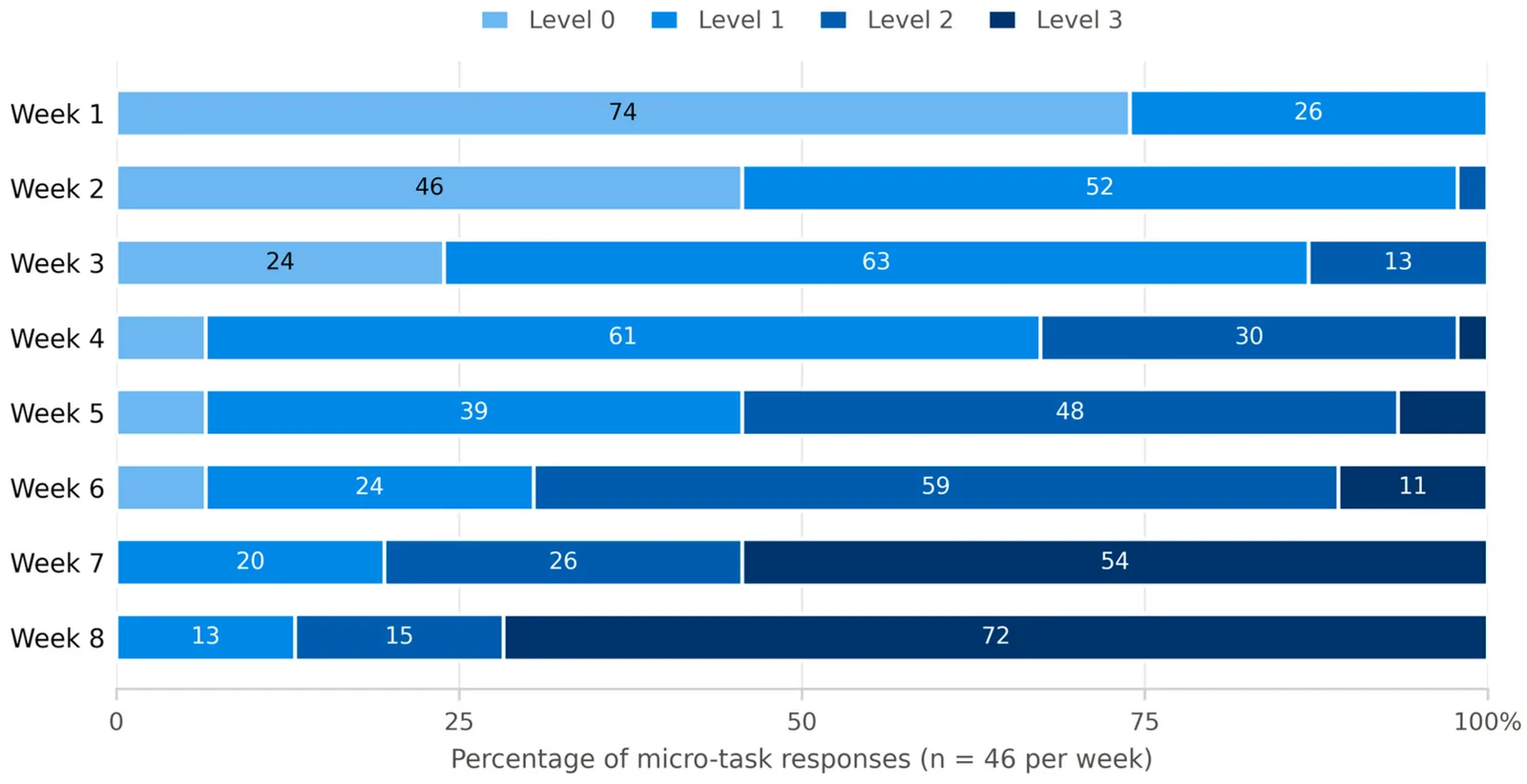
Rubric levels (0–3) in the eight weekly micro-tasks of the experimental group (*n* = 46 per week). Each task addressed a different concept (Table A4), so the series is not a repeated measure.

The 46 experimental-group students produced 368 weekly micro-task responses. In Week 1, 73.9% were scored at Level 0 and none at Levels 2 or 3. Figure 3 shows the full distribution across weeks. Students often classified a system as AI because it moved, used a sensor, or “worked by itself.” By the middle weeks, explanations increasingly referred to mechanism: “It does the same thing every time. We already told it what to do.” During classification activities, students described learning from examples: “We show it the movements first, then it tries to find which one the new movement looks like.” They also separated input from assigned meaning: “It does not really see the plastic. It sees the movement I make.”

Advanced explanations became most visible in the data-diversity and final design activities. In Week 7, 54.3% of responses reached Level 3. Students linked misclassification with the limits of the training set and began to distinguish quantity from variety: “Different examples are more important than just lots of examples because everyone is different.” In Week 8, 71.7% reached Level 3. Many students evaluated technology–problem fit: “If it only needs to turn on when someone comes, you do not need AI” and “If a simple rule is enough, AI is not necessary.”

The thematic analysis produced six connected patterns. First, students moved from appearance-based labels toward distinguishing learned classification from fixed rules. Interview and worksheet evidence increasingly referred to whether the system had been trained rather than whether it was robotic or automatic. Second, they learned to identify the actual input. In the recycling task, early descriptions claimed the model recognized plastic or paper; later explanations recognized that it classified a body pose linked by people to those categories.

Third, students began to explain error through training data and user variation. Early debugging focused on repeating a movement or changing code. Later, students inspected which examples had been included, tested with different children, and added variation in distance, angle, lighting, and movement. Statements such as “If it only saw children in our group, it might get confused when another child comes” reflected this shift.

Fourth, model error led to more qualified trust. Students did not simply reject AI after observing failure. They proposed retesting and human review, especially when the consequence mattered: “If it is something important, a person should check too.” Fifth, the accessibility activity changed the criterion of success from whether a system worked to whether different users could use it. “Not everyone can squat” prompted alternatives such as sound, buttons, or simpler movements. Some groups incorporated or proposed these alternatives in their redesigned systems.

Sixth, testing and redesign became a source of conceptual learning. Students described moving from “it is broken” toward locating the reason for unexpected behavior. By the final weeks, testing addressed not only code correctness but also data coverage, user differences, system limitations, and whether AI had been an appropriate choice. Statements such as “Before, I thought every robot was AI” and “Now I first think about what it really recognizes” captured the broader conceptual change.

Table 5 links these patterns to the assessment domains by juxtaposing a less advanced and a more advanced response in each domain; Figure 3 shows how common each level was in each week.

### 4.7. Integration of Findings

The quantitative and qualitative evidence converged. The large adjusted AI literacy difference was accompanied by gains across all eight domains, movement into data-based and critical profiles, and increasingly mechanistic explanations. The domain with the greatest improvement—data diversity and different users—was also central in observations of model failure and retraining. The rise in computational thinking corresponded with repeated decomposition, sequencing, testing, debugging, and redesign. At the same time, the nonsignificant within-group correlations supported the interpretation that AI literacy and computational thinking were mutually useful but distinct: programming practices did not fully account for students’ reasoning about data, oversight, accessibility, or AI necessity.

## 5. Discussion

### 5.1. AI Literacy Gains and the Role of Contrast (RQ1)

The intervention produced significant development in both AI literacy and computational thinking, but its most important contribution lies in the form of understanding students developed. They moved beyond identifying AI through robots, sensors, and automatic behavior and began to explain systems through rules, training examples, classification, input, and error. This addresses a recurring problem in primary AI education: children can use the language of intelligence while relying on visible features rather than system mechanisms (Walan, 2025; Yim, 2024). The findings show that third-grade students can begin to build more precise mental models when machine learning is placed beside a clearly contrasting non-AI system.

The magnitude of the AI literacy effect should be read in relation to this close alignment among curriculum, activity, and assessment. Students encountered the target ideas repeatedly, expressed them in different problem contexts, and were assessed through scenarios that required the same kinds of explanation and judgment. The result does not imply that brief exposure to any AI platform will produce comparable gains. Reviews of K–12 AI education continue to report substantial variation in content, instructional time, learner age, and assessment quality (Lee & Kwon, 2024; Liu & Zhong, 2024; Martin et al., 2024). What the present findings support more specifically is the value of a coherent sequence in which core concepts are revisited through construction, comparison, testing, and reflection.

The contrastive design was central. Students learned what AI was partly by examining what it was not. A sensor-based warning system and a trained pose classifier could both produce automatic responses, but only one learned a classification from examples. Repeated comparison made the decision mechanism discussable. This extends earlier work showing that machine learning concepts can be introduced in elementary settings through construction and experimentation (Shamir & Levin, 2022). Rather than presenting AI as a single category of “smart” technology, the intervention organized learning around evidence for classifying a system.

### 5.2. Reasoning About Input, Data, Error, Oversight, Accessibility, and AI Necessity (RQ3)

A second contribution concerns actual input. In the recycling activity, the classifier did not recognize a recyclable material; it recognized a pose that students had assigned to a category. Understanding this distinction is foundational because users often attribute human-level meaning to an AI output. The movement from “it sees plastic” to “it sees my movement” shows that young learners can separate the technical input from the interpretation imposed by designers. Data-related conceptions of this kind are increasingly recognized as essential to K–12 AI education (Olari & Romeike, 2024), yet they are often less visible than tool operation.

Data diversity and model error provided the strongest evidence of explanatory development. Students saw a model perform well for users and conditions resembling its training examples and fail under changed conditions. Retraining with more varied examples turned an abstract statement—diverse data matter—into an observable relationship between design choices and behavior. The distinction some students drew between many similar examples and varied examples is especially notable. It represents an early form of reasoning about representation and generalization, even though posttest performance showed that the idea remained demanding.

This process also grounded inclusion in technical experience. When a model worked better for students represented in the training data, questions of fairness were no longer detached ethical topics. Students could ask who supplied the examples, for whom the system worked, and who was missing. Reviews of responsible AI education emphasize the need to connect ethical awareness with action and mitigation (Ma et al., 2025). In the present study, adding users, changing inputs, and redesigning the interface were concrete responses to limitations students had observed themselves.

Model failure also supported more realistic trust. Students initially treated incorrect output as evidence that the product was broken. Later, they examined code, rules, labels, examples, and user variation before deciding what to change. Human oversight emerged from this inquiry. Students increasingly argued that an important decision should be checked, not because all AI is unreliable, but because a model can be wrong under specific conditions. This is a developmentally appropriate route into accountability: the system produces an output, but people remain responsible for testing, interpreting, and acting on it.

Accessibility broadened the meaning of technical success. A gesture-controlled system could function accurately and still be inappropriate for users who could not perform the required pose. Students’ proposals for buttons, sound, or alternative movements show that primary learners can treat user diversity as a design requirement. Multidimensional AI literacy frameworks include social and ethical considerations (Ng et al., 2021; Yim & Su, 2025), but the current findings show how those considerations can be embedded inside construction and redesign rather than added as a separate discussion after the product is complete.

The most distinctive finding was the development of judgment about non-use. At pretest, deciding whether AI was necessary was the weakest domain. Many students initially preferred AI because it appeared more advanced. By the final week, they could justify rule-based systems when the problem required only a predictable response. This extends critical AI literacy beyond evaluating a system after it has been introduced. It treats the decision to select or reject AI as part of the literacy itself.

Problem–technology fit matters particularly in integrated STEM. Good integration is not achieved by inserting the largest possible number of technologies into an activity. It depends on whether disciplinary knowledge, computational practices, and design choices serve the problem. A fixed rule may be more transparent, reliable, economical, and controllable than a classifier. Recognizing this is not a lack of ambition; it is evidence of technological judgment. The finding responds directly to the Special Issue’s concern with how AI literacy reshapes STEM thinking: students did not merely learn with AI but learned to question its value in a STEM solution.

### 5.3. Computational Thinking, Its Relation to AI Literacy, and Design Practices (RQ2 and RQ4)

The computational thinking result supports the value of iterative design but should not be attributed to the LEGO environment alone. Students repeatedly decomposed problems, ordered actions, wrote conditions, tested behavior, located discrepancies, and revised systems. The positive effect is consistent with research showing that structured programming and debugging can support computational thinking in primary education (Falloon, 2024; Misirli & Komis, 2023). It also helps explain why robotics research produces variable outcomes. Ouyang and Xu (2024) found that educational robotics does not automatically yield substantial computational thinking gains; instructional strategy, duration, and interaction matter. In this study, the physical kit was the medium for a recurring reasoning cycle, supported by teacher prompts and role rotation.

The correlation findings clarify the relationship between the two outcomes. Across all students, gains were related, but the association disappeared within each condition. The overall correlation was partly created by the fact that experimental-group students improved more on both measures. AI literacy and computational thinking intersected in practice, yet individual gains did not rise together in a uniform way. Computational thinking supported construction and debugging, whereas AI literacy required additional reasoning about training, actual input, representation, oversight, accessibility, and appropriateness. Curriculum and assessment should therefore specify and measure both rather than using computational performance as a proxy for AI understanding.

The Life Studies contexts strengthened the intervention by making technical choices consequential. Safety, recycling, energy, traffic, and accessibility gave students reasons to define users, examine failure, and compare alternatives. This supports interdisciplinary approaches that connect AI with existing subjects through data, models, evidence, and design (Park et al., 2023). The context did more than increase engagement: it shaped the questions students could ask. Human oversight mattered in a safety problem; accessibility mattered in an assistance system; AI necessity could be judged because a real problem had been defined.

### 5.4. Methodological Implications

The assessment approach also contributed to the findings. Open-ended performance tasks revealed whether students could justify a classification or design choice rather than merely report confidence. Weekly micro-tasks and profiles showed that development involved a reorganization of explanations, not only a higher total score. The profiles should not yet be treated as a universal developmental sequence, and the instrument requires further validation. Even so, combining performance measures with process evidence offered a more informative account than relying on a self-report scale or a successful group product alone.

## 6. Conclusions

Primary students can develop critical AI literacy when instruction makes system mechanisms observable, treats error as evidence, and compares AI with simpler alternatives. The experimental group showed substantial gains in AI literacy and computational thinking, but the qualitative evidence is equally important: students learned to ask what a system recognizes, how training examples shape behavior, why it fails for some users, when a person should check the result, and whether AI adds value.

For curriculum design, AI and rule-based automation should be taught comparatively. Training data should be made visible through testing with different users and conditions. Technical activities should include human-centered questions from the outset, and teachers should normalize the conclusion that AI is unnecessary when a fixed rule solves the problem more appropriately. Performance tasks should assess explanations and decisions, while computational thinking and AI literacy should remain separate outcomes even when taught together. The domain results suggest priorities: most students learned to distinguish AI from rules and to explain training within eight weeks, whereas AI necessity, data quantity versus diversity, and accessibility remained demanding and deserve more time and explicit assessment.

Teacher preparation should extend beyond platform operation. Teachers need working knowledge of machine learning, fixed-rule systems, input, classification, data quality, model limitations, accessibility, and oversight. They also need prompts that expose student reasoning without immediately supplying the answer. Questions such as “What does the system actually recognize?”, “Did it learn or follow a rule?”, “Why did it fail for another user?”, and “Could a simpler system solve the problem?” can turn design activity into conceptual inquiry. Because the intervention was delivered by an experienced researcher, transferring it to regular classrooms will require professional development that covers both the kit and these conceptual questions.

At the policy and curriculum level, the findings support a shift from exposure-based targets toward observable reasoning outcomes. Curriculum statements should specify that students compare AI with non-AI systems, explain the role of examples and labels, test across users and contexts, and evaluate the need for human judgment. AI literacy should also be integrated with, but not absorbed into, computational thinking. Such an approach is consistent with calls for primary AI education that is interdisciplinary, developmentally appropriate, and attentive to social as well as technical understanding (Atias & Mawasi, 2025; Stolpe & Hallström, 2024; Yang, 2022; Yim & Su, 2025).

## 7. Limitations and Future Research

The findings should be interpreted within the study’s boundaries. Participants came from four intact classrooms in one school, individual random assignment was not possible, and the small number of classroom clusters prevented robust multilevel modeling. Unmeasured classroom or teacher differences may therefore have contributed to the results. The control condition was business-as-usual rather than an active comparison, so the contribution of the AI–automation contrast cannot be separated from that of additional technology-rich instruction, and instruction was delivered by the second author rather than the classroom teachers, so the results reflect expert facilitation. The intervention lasted eight weeks and did not include a delayed posttest, so retention and transfer to everyday commercial AI remain unknown.

The machine learning activities focused mainly on pose classification. This made training and error visible but does not represent language, recommendation, generative, or multimodal systems. The AI Literacy Performance Assessment was developed for this study and closely aligned with the intervention. Although expert review, pilot work, parallel forms, and explicit scoring strengthened it, broader psychometric validation is needed. Formal inter-rater and inter-coder agreement coefficients were not computed; future applications should include independent double scoring of a random subset of responses. Weekly tasks also differed in content and difficulty, so their distributions indicate process rather than a linear growth scale. Collaborative products may reflect unequal participation despite role rotation, and the novelty of the materials may have influenced motivation.

Future research should use larger multisite samples and more classroom clusters, include active comparison conditions, examine implementation by classroom teachers after professional development, and add delayed and transfer assessments. Comparative designs could separate the effects of robotics, AI–automation contrast, authentic STEM problems, and iterative redesign. Studies should examine whether students apply the same reasoning to image, sound, text, recommendation, and generative systems. Further work is also needed to validate the performance assessment and profiles and to identify the teacher knowledge and discourse practices that best support mechanistic, critical, and human-centered AI literacy.

## Figures and Tables

**Table 1:** Overview of the eight-week intervention.

Week	Context and System	Student Task	Primary Conceptual Focus
1	Smart and safe school; rule-based warning system	Choose a school-safety problem, code an event- or sensor-triggered warning, and test it in different situations	Automatic action and sensor use do not necessarily indicate AI
2	AI-supported movement coach; pose classifier	Define three or four poses, link each to a LEGO response, train the classifier, and test it with different students	Training examples, labels, classification, and prediction
3	Recycling game through body poses; pose classifier	Assign a pose to each recycling category, train the classifier, and explain what the model actually recognizes	Actual model input versus the meaning assigned by users
4	Energy-saving classroom; rule-based automation	Write fixed if–then rules for an energy-saving device and test them in at least three situations	Predefined conditions versus learning from examples
5	Safe pedestrian crossing; pose classifier	Train a crossing-request classifier with examples from different students and compare its results for four users	Testing across users and conditions
6	Accessible assistance system; AI and alternative inputs	Design a pose-based assistance system for a user profile and propose alternatives for users who cannot perform the poses	Accessibility, user diversity, and human-centered design
7	Why does AI make mistakes?; retrained classifier	Train with limited data, test with new users, retrain with varied data, and compare the two models	Data diversity, representativeness, model error, and revision
8	AI or automation solution for society	Design an AI or rule-based solution to a real community problem and justify the choice	Problem–technology fit, oversight, limitations, and AI necessity

**Table 2:** Pretest and posttest scores by group.

Outcome	Group	Pretest M (SD)	Posttest M (SD)	Pre–Post t (df)	*p*	d	Adjusted Posttest M
AI literacy	Experimental (*n* = 46)	8.85 (5.57)	18.04 (6.16)	16.39 (45)	<.001	2.42	17.33
AI literacy	Control (*n* = 47)	7.36 (5.97)	8.85 (6.30)	5.22 (46)	<.001	0.76	9.55
Computational thinking	Experimental (*n* = 46)	10.57 (4.94)	14.24 (4.90)	7.73 (45)	<.001	1.14	14.74
Computational thinking	Control (*n* = 47)	11.79 (4.56)	12.02 (5.17)	0.46 (46)	.648	0.07	11.53

Note. Pre–post t = paired-samples *t* test within each group; d = Cohen’s d for paired data. Adjusted posttest means are from ANCOVAs with the corresponding pretest as covariate: AI literacy, F(1, 90) = 152.51, *p* < .001, partial η^2^ = 0.629; computational thinking, F(1, 90) = 22.18, *p* < .001, partial η^2^ = 0.198.

**Table 3:** Change across the eight AI literacy domains by group.

Domain	Group	Pretest M (SD)	Posttest M (SD)	t	*p*	d	Gain t	*p*	d
AI versus rule-based systems	Experimental	1.80 (0.96)	2.76 (0.60)	7.96	<.001 *	1.17	4.20	<.001 *	0.87
	Control	1.51 (1.06)	1.77 (1.05)	2.21	.032	0.32			
Training data and classification	Experimental	1.43 (0.86)	2.61 (0.77)	10.35	<.001 *	1.53	6.81	<.001 *	1.41
	Control	1.43 (0.99)	1.55 (0.93)	1.23	.224	0.18			
Actual input recognized by AI	Experimental	1.28 (0.91)	2.50 (0.86)	10.50	<.001 *	1.55	7.18	<.001 *	1.49
	Control	1.11 (0.98)	1.17 (0.99)	0.57	.569	0.08			
Data diversity and different users	Experimental	1.17 (0.90)	2.41 (0.80)	9.91	<.001 *	1.46	6.18	<.001 *	1.29
	Control	0.98 (0.85)	1.23 (0.94)	2.59	.013	0.38			
AI errors and human oversight	Experimental	0.98 (0.88)	2.11 (0.92)	8.92	<.001 *	1.32	6.49	<.001 *	1.35
	Control	0.79 (0.88)	0.96 (0.93)	2.23	.031	0.32			
Human-centered AI and accessibility	Experimental	0.89 (0.88)	2.00 (0.97)	9.14	<.001 *	1.35	5.74	<.001 *	1.19
	Control	0.64 (0.79)	0.87 (0.92)	2.54	.015	0.37			
Data quantity versus diversity	Experimental	0.72 (0.78)	1.89 (1.06)	8.60	<.001 *	1.27	6.37	<.001 *	1.32
	Control	0.57 (0.71)	0.68 (0.81)	1.09	.280	0.16			
Whether AI is necessary	Experimental	0.57 (0.69)	1.76 (1.08)	8.27	<.001 *	1.22	5.49	<.001 *	1.14
	Control	0.34 (0.76)	0.62 (0.77)	3.28	.002 *	0.48			

Note. Within-group columns report paired *t* tests (df = 45 experimental, 46 control) with Cohen’s d for paired data; gain columns compare pre–post gains between groups (Welch’s t, Cohen’s d). * significant at the Bonferroni-adjusted α = 0.006. Wilcoxon and Mann–Whitney tests led to the same conclusions.

**Table 4:** AI competency profiles before and after the intervention.

Group	Profile	Pretest *n* (%)	Posttest *n* (%)
Experimental	Technology-centered	19 (41.3)	5 (10.9)
Experimental	Functional	18 (39.1)	3 (6.5)
Experimental	Data- and learning-based	9 (19.6)	17 (37.0)
Experimental	Critical and human-centered	0 (0.0)	21 (45.7)
Control	Technology-centered	28 (59.6)	21 (44.7)
Control	Functional	10 (21.3)	15 (31.9)
Control	Data- and learning-based	7 (14.9)	8 (17.0)
Control	Critical and human-centered	2 (4.3)	3 (6.4)

**Table 5:** Illustrative less and more advanced student responses by AI literacy domain (experimental group).

Domain	Less Advanced Response (Source; Level)	More Advanced Response (Source; Level)
AI versus rule-based systems	“Because it moved.” (W1; 0)	“The sensor only provides input. If the system reacts according to a fixed rule, it is automation; if it learns from examples and makes predictions, it may be AI.” (W4; 3)
Training data and classification	“The camera looks at my body and guesses.” (W2; 1)	“First we give it examples of different poses. Then it matches the new pose to one of the classes it has learned.” (interview)
Actual input recognized by AI	“It recognizes the waste.” (W3; 0)	“It does not see the waste itself; it sees the movement I make.” (W3; 2)
Data diversity and different users	“Sometimes it may not recognize it.” (W5; 1)	“If the AI was trained only with some students’ examples, it may misrecognize other people.” (W5; 3)
AI errors and human oversight	“It could not look well enough.” (W7; 1)	“The AI’s output should not be accepted as correct right away; it should be retested … and especially for important decisions there should be human oversight.” (interview)
Human-centered AI and accessibility	“It helps everyone.” (W6; 0)	“… not everyone may be able to make the same movement. There should be options such as another movement, a button, or sound.” (W6; 3)
Data quantity versus diversity	“If we show it more examples, it may improve.” (W7; 2)	“What matters is not more examples but more varied ones; there should be different people, angles, and distances.” (W7; 3)
Whether AI is necessary	“Maybe automation could work too.” (W8; 1)	“If the problem only requires a response under a specific condition, automation is enough. If new poses have to be learned from examples and classified, AI makes sense.” (W8; 3)

Note. Responses translated from Turkish. W = week of the micro-task (Table A4); the number after the semicolon is the rubric level; interview responses were not scored.

## Data Availability

The raw data supporting the conclusions of this article will be made available by the authors, without undue reservation.

## Supplementary Materials

The following supporting information can be downloaded at: https://www.mdpi.com/article/10.3390/jintelligence14100257/s1, File S1: AI Literacy Performance Assessment, Forms A and B, with scoring rubric and scorer notes (English translation and original Turkish version); File S2: Teacher implementation guide (English translation and original Turkish version).

## Appendix A

The intervention followed a written implementation guide (English translation and Turkish original in File S2). Each kit was shared by a team of three or four students, who rotated four roles weekly (design and modeling; coding and algorithm; data and AI; testing and improvement). The instructor presented the problem, asked neutral questions (e.g., “Does the system learn from new examples?”, “What happens if another student makes the same movement?”, “Could a simpler solution be enough?”), and avoided statements that would give away conclusions (e.g., “This system is not AI”, “You should collect data from different students”). Table A1 gives the suggested weekly time plan, Table A2 and Table A3 the plans for Weeks 1 and 7, Table A4 the micro-task questions, and Table A5 the focus-group questions. All materials were translated from Turkish.

**Table A1:** Suggested weekly time plan (minutes).

Phase	Minutes
Presentation and discussion of the problem	10–15
Problem selection and decomposition	10–15
Algorithm or solution plan	10–15
LEGO design and coding	35–45
Training (AI weeks) and testing	15–20
Debugging and redesign	15–20
Teamwork form	10
Individual micro-task	10

Note. Durations are the ranges suggested in the implementation guide. They were adapted to each week’s activity (training took place only in AI weeks), and each week’s activity took about 105–135 min in total, delivered in two sessions.

**Table A2:** Plan for Week 1: Smart and safe school (rule-based pathway).

Component	Description
Life Studies context	Me and My School; My Health and Safety
Core problem	Can we design a system that notices a safety problem at school and warns the student?
Technical structure	Events; simple conditions; input–process–output; sensor or event–response. No AI is used.
Opening questions	Which situations at school can be dangerous? How could a system notice the danger? What should it do then?
Steps	Students list school-safety problems; each team chooses one, decomposes it, orders the operations, builds and codes the model, tests it in different situations, and redesigns it if needed.
Critical discussion	Is the system you designed really AI? Neutral prompts: Does it learn from new examples, or follow the rule you gave it? Does using a sensor make a system AI?
Instructor must not say	“This is not AI.” Students are expected to reach this conclusion themselves.
Micro-task	“Was the system you designed really artificial intelligence? Why?”

**Table A3:** Plan for Week 7: Why does AI make mistakes? (machine learning pathway).

Component	Description
Life Studies context	Science, Technology and Art; possibilities and limitations of technology
Core problem	Why does AI sometimes recognize the same movement correctly and sometimes incorrectly?
Phase 1: limited data	Train the classifier with one or a few students, a similar angle and distance, and few examples; test it with different students.
Phase 2: varied data	Retrain with different students, distances, and small variations in the pose; compare the two models.
Neutral prompts	Which examples was the first model trained with? When did it fail? Could many similar examples from one person be enough? What might examples from different people change?
Instructor must not say	“If you use more varied data, the model will work better.”
Micro-task	“Why did the AI make a mistake, and what kind of training data would it need to work more accurately?”

**Table A4:** Weekly micro-task questions (answered individually, scored 0–3).

Week	Question
1	Was the system you designed really artificial intelligence? Why?
2	How might the AI be recognizing the pose you make?
3	Does this system really recognize the waste, or your body pose? Explain.
4	Is every system that uses a sensor artificial intelligence? Explain with an example.
5	Can the AI recognize everyone who makes the same pose with the same accuracy? Why?
6	Whom does the system you designed help, and in which situation might it fall short?
7	Why did the AI make a mistake, and what kind of training data would it need to work more accurately?
8	Was AI really necessary for this problem? Could a simpler automation have been enough?

**Table A5:** Focus-group interview questions.

No	Question
1	What is the most important difference between AI and a system that works automatically?
2	How might AI learn a body pose?
3	Why might AI give different results when different students make the same pose?
4	Which is more useful: many identical examples or fewer but different examples? Why?
5	What should be done when AI makes a mistake?
6	Could an AI system be unsuitable for some people? Why?
7	Is it necessary to use AI for every real-life problem?
8	What was the biggest change in your thinking about AI over the eight weeks?

## Appendix B

Table A6 reproduces the two parallel forms as administered (translated from Turkish; the original forms and scorer notes are provided in File S1). Students read each scenario or had it read aloud, and each response was scored with the common rubric in Table A7 (total score 0–24).

**Table A6:** AI Literacy Performance Assessment: Form A (pretest) and Form B (posttest).

Domain	Form A (Pretest)	Form B (Posttest)
1. AI versus rule-based systems	A motion sensor has been installed in the school corridor. When the sensor notices that a student is passing, the light turns on automatically. The system was given the command “if there is movement, turn on the light” in advance.—Do you think this system is artificial intelligence? Why do you think so?	The irrigation system in a school garden starts automatically every morning at 08:00. The system was given the command “if it is 08:00, start watering” in advance.—Do you think this system is artificial intelligence? Why?
2. Training data and classification	A computer was first shown many “hands up” and “hands down” poses. Later, the computer looked at the pose of a student it had never seen before and said that the student belonged to the “hands up” group.—How might the computer have guessed which group the new student belonged to? Can you explain?	A computer was shown many “leaning to the right” and “leaning to the left” movements beforehand. Then the computer identified the movement of a student it had never seen before as “leaning to the right”.—How might the computer have decided which group this new movement belongs to?
3. Actual input recognized by AI	A group of students prepared a recycling game. Raising the arms means “paper”, opening the hands to the sides means “plastic”, and squatting means “glass”. The camera looks at the student’s movement and turns on the light of the correct recycling bin.—Do you think the computer really sees the paper, the plastic, and the glass? Or does it see something else? Explain.	Students created a traffic game. Hands above the head means “stop”, and hands opened to both sides means “go”. After the camera recognizes the movement, the LEGO traffic light turns red or green.—Do you think the AI sees the ideas of “stop” and “go”, or does it recognize something else? Can you explain?
4. Data diversity and different users	An AI system was trained using only the hand movements made by Ece. The system recognizes Ece’s movements correctly. Later, when Mehmet makes the same movements, the system sometimes gives wrong answers.—What do you think the reason could be? What could be done so that the system recognizes Mehmet better, too?	A movement-recognition system was trained using the movements of only three tall students. Later, when shorter students tried the system, some movements were misrecognized.—Why do you think the system may have made mistakes? What should be changed during training so that it works better?
5. AI errors and human oversight	At a school, an AI-supported system opens the door by recognizing the movement a student makes. One day, although the student made the correct movement, the system misrecognized it and did not open the door.—Why do you think the AI might have made a mistake? In such a situation, is it right to trust only the AI’s decision? Why?	An AI system misunderstood a student’s help gesture and answered, “no help needed”. The student, however, really needs help.—Why might this system have made a mistake? In such an important situation, should a person also check the AI’s decision? Why?
6. Human-centered AI and accessibility	A help system calls for help when a person raises both arms at once. However, there is also a student at the school who has difficulty raising their arms.—Do you think this system is suitable for everyone? How could you change it so that this student can use the system, too?	In a school game, the child has to squat so that the AI recognizes the student. A student who uses a wheelchair cannot make this movement.—Do you think the game is fair and usable for this student? How could you change the system so that everyone can use it?
7. Data quantity versus diversity	Two groups prepared AI systems that recognize the same movement. The first group showed the same student’s same movement 100 times. The second group used 40 examples, but collected them from different students, from different distances, and with slightly different forms of the movement.—Which system do you think can recognize a new student’s movement better? Why?	One group showed an AI system 80 movement examples from the same student, in the same room and from the same distance. Another group collected 50 examples from different students, from different distances, and from slightly different angles.—Which system do you think may be more successful when a student it has never seen before arrives? Why?
8. Whether AI is necessary	In a classroom, the lamps should turn on automatically when it gets dark. The students proposed two solutions. The first is to use a light sensor that turns on the lamp when the light decreases. The second is to develop an AI system for this.—Which solution would you choose? Is it necessary to use AI for this problem? Why?	The school entrance door should open only when a button is pressed. One student proposed a simple system in which the button opens the door, and another student wanted to use AI for this.—Which would you choose? Is AI necessary here? Why?

**Table A7:** Common scoring rubric applied to every task.

Score	Criterion
0	No response, the response is irrelevant to the question, or the core concept is explained entirely incorrectly.
1	The student explains the situation through surface features, using technology-centered justifications such as “it is AI because it is a robot”, “it is AI because it does it by itself”, or “because the computer knows”.
2	The core concept is understood correctly, but the explanation is incomplete. The student uses at least one relevant concept—learning from examples, rules, data, error, or user differences—correctly but cannot fully explain the relationships among them.
3	The student explains the mechanism correctly and justifies the answer. Depending on the question, the student clearly and consistently relates learning from examples to rules, the actual input to the assigned meaning, data diversity to performance, AI error to human control, or the need for the technology to the problem.
