# Supplementary material for: When AI Is—and Is Not—Needed: AI Literacy and Computational Thinking in Primary STEM
Source: J Intell. 2026 Oct 12;14(10):257. doi: 10.3390/jintelligence14100257 (PMC13706255; doi:10.3390/jintelligence14100257)
Supplement: Supplementary file 1 [file jintelligence-14-00257-s001.zip › Supplementary_File_S1_AI Literacy Performance Assessment_Forms A and B_with scoring rubric and scorer notes_EN_TR.pdf]

Supplementary File S1

AI Literacy Performance Assessment for Primary School Students

Form A (Pretest) and Form B (Posttest) with the Common Scoring Rubric and Scorer Notes

This file provides the two parallel forms of the AI Literacy Performance Assessment used at pretest (Form A) and posttest (Form B) in the study "When AI Is—and Is Not—Needed: AI Literacy and Computational Thinking in Primary STEM", together with the common scoring rubric and the task-specific notes for scorers. Part 1 is an English translation; Part 2 reproduces the original Turkish version administered to the students. The forms and the rubric also appear in Appendix B (Tables A6 and A7) of the article.

Part 1. English Translation

Instructions for Students

In this activity, you will meet some everyday situations. Read each situation carefully or listen to your teacher. The questions may not have one-word answers. It is important to explain what you think and why you think so. Do not be afraid of giving a wrong answer. Here, we are trying to understand how you think.

Each question will be scored from 0 to 3. The total score is 24.

FORM A – Pretest

Question 1 – Artificial intelligence or automation?

A motion sensor has been installed in the school corridor. When the sensor notices that a student is passing, the light turns on automatically. The system was given the command "if there is movement, turn on the light" in advance.

Do you think this system is artificial intelligence? Why do you think so?

Answer:

Question 2 – Learning from examples and classification

A computer was first shown many "hands up" and "hands down" poses. Later, the computer looked at the pose of a student it had never seen before and said that the student belonged to the "hands up" group.

How might the computer have guessed which group the new student belonged to? Can you explain?

Answer:

Question 3 – What does the AI actually see?

A group of students prepared a recycling game. Raising the arms means "paper", opening the hands to the sides means "plastic", and squatting means "glass". The camera looks at the student's movement and turns on the light of the correct recycling bin.

Do you think the computer really sees the paper, the plastic, and the glass? Or does it see something else? Explain.

Answer:

Question 4 – Different people and data diversity

An AI system was trained using only the hand movements made by Ece. The system recognizes Ece's movements correctly. Later, when Mehmet makes the same movements, the system sometimes gives wrong answers.

What do you think the reason could be? What could be done so that the system recognizes Mehmet better, too?

Answer:

Question 5 – AI error and human control

At a school, an AI-supported system opens the door by recognizing the movement a student makes. One day, although the student made the correct movement, the system misrecognized it and did not open the door.

Why do you think the AI might have made a mistake? In such a situation, is it right to trust only the AI's decision? Why?

Answer:

Question 6 – Can everyone use it?

A help system calls for help when a person raises both arms at once. However, there is also a student at the school who has difficulty raising their arms.

Do you think this system is suitable for everyone? How could you change it so that this student can use the system, too?

Answer:

Question 7 – Many examples or different examples?

Two groups prepared AI systems that recognize the same movement. The first group showed the same student's same movement 100 times. The second group used 40 examples, but collected them from different students, from different distances, and with slightly different forms of the movement.

Which system do you think can recognize a new student's movement better? Why?

Answer:

Question 8 – Is AI really necessary?

In a classroom, the lamps should turn on automatically when it gets dark. The students proposed two solutions. The first is to use a light sensor that turns on the lamp when the light decreases. The second is to develop an AI system for this.

Which solution would you choose? Is it necessary to use AI for this problem? Why?

Answer:

FORM B – Posttest

Form B assesses the same eight competencies; however, the contexts were changed to reduce the likelihood that students answer by remembering the Form A scenarios.

Question 1 – Artificial intelligence or automation?

The irrigation system in a school garden starts automatically every morning at 08:00. The system was given the command "if it is 08:00, start watering" in advance.

Do you think this system is artificial intelligence? Why?

Answer:

Question 2 – Learning from examples and classification

A computer was shown many "leaning to the right" and "leaning to the left" movements beforehand. Then the computer identified the movement of a student it had never seen before as "leaning to the right".

How might the computer have decided which group this new movement belongs to?

Answer:

Question 3 – What does the system actually detect?

Students created a traffic game. Hands above the head means "stop", and hands opened to both sides means "go". After the camera recognizes the movement, the LEGO traffic light turns red or green.

Do you think the AI sees the ideas of "stop" and "go", or does it recognize something else? Can you explain?

Answer:

Question 4 – Encountering new users

A movement-recognition system was trained using the movements of only three tall students. Later, when shorter students tried the system, some movements were misrecognized.

Why do you think the system may have made mistakes? What should be changed during training so that it works better?

Answer:

Question 5 – Error and human oversight

An AI system misunderstood a student's help gesture and answered, "no help needed". The student, however, really needs help.

Why might this system have made a mistake? In such an important situation, should a person also check the AI's decision? Why?

Answer:

Question 6 – Accessible design

In a school game, the child has to squat so that the AI recognizes the student. A student who uses a wheelchair cannot make this movement.

Do you think the game is fair and usable for this student? How could you change the system so that everyone can use it?

Answer:

Question 7 – Data quantity and diversity

One group showed an AI system 80 movement examples from the same student, in the same room and from the same distance. Another group collected 50 examples from different students, from different distances, and from slightly different angles.

Which system do you think may be more successful when a student it has never seen before arrives? Why?

Answer:

Question 8 – Is it necessary to use AI?

The school entrance door should open only when a button is pressed. One student proposed a simple system in which the button opens the door, and another student wanted to use AI for this.

Which would you choose? Is AI necessary here? Why?

Answer:

Common Scoring Rubric

Score	Criterion
0	No response, the response is irrelevant to the question, or the core concept is explained entirely incorrectly.
1	The student explains the situation through surface features, using technology-centered justifications such as "it is AI because it is a robot", "it is AI because it does it by itself", or "because the computer knows".
2	The core concept is understood correctly, but the explanation is incomplete. The student uses at least one relevant concept—learning from examples, rules, data, error, or user differences—correctly but cannot fully explain the relationships among them.

Score	Criterion
3	The student explains the mechanism correctly and justifies the answer. Depending on the question, the student clearly and consistently relates learning from examples to rules, the actual input to the assigned meaning, data diversity to performance, AI error to human control, or the need for the technology to the problem.

With each of the eight tasks in a form scored from 0 to 3, the total score ranges from 0 to 24.

Scorer Notes: The Competency Assessed by Each Task

Task	Form A	Form B
1	This question assesses whether the student regards automatic operation alone as AI and whether the student understands the difference between a predetermined rule and learning.	A conceptually correct explanation is expected to state that automatic operation alone does not mean AI and that the system applies a predefined rule.
2	This item assesses whether the student sees AI as a system that "knows by itself" or understands that it classifies new input by drawing on the examples it was given beforehand.	This task assesses the same understanding of learning and classification as the second task of Form A, through a different scenario.
3	This question assesses whether the student can distinguish the actual input processed by the system from the meaning that people assign to that input.	A high-level response should explain that the system classifies body poses and that the meanings "stop" and "go" were assigned to the categories by people.
4	This item examines whether the student recognizes that training data need to include different people and different conditions.	This question assesses whether the student can generalize the idea of data diversity to the context of different user characteristics.
5	This task assesses both the explanation of model error and the need for human control.	In addition to model error, this task assesses reasoning about human oversight in situations where the consequences of an error are important.
6	This question assesses understanding of accessibility and human-centered design.	This item assesses not only whether the student notices the problem but also whether the student proposes an alternative, more inclusive design.
7	This item assesses whether the student attends only to the quantity of data or also to data diversity.	This task assesses whether the student can move beyond the idea that "more data is always better" and explain the importance of data diversity for generalization.
8	This task assesses whether the student regards AI as the preferred technology for every problem and whether the student can justify the technological fit of a solution.	The most advanced response is expected not merely to say that AI could be used, but to justify that solving the problem does not require learning or classifying variable inputs and that a simple, explicit rule is sufficient.

Part 2. Original Turkish Version (Özgün Türkçe Sürüm)

İlkokul Öğrencileri İçin Yapay Zekâ Okuryazarlığı Performans Değerlendirmesi

Form A (Ön Test) ve Form B (Son Test) — Özgün Türkçe Sürüm ve Ortak Puanlama Anahtarı

Öğrenci Yönergesi

Bu çalışmada bazı günlük yaşam durumlarıyla karşılaşacaksın. Her durumu dikkatlice oku veya öğretmenini dinle. Soruların tek bir kelimelik cevabı olmayabilir. Ne düşündüğünü ve neden böyle düşündüğünü açıklaman önemlidir. Yanlış cevap vermekten korkma. Burada senin düşünme biçimini anlamaya çalışıyoruz.

Her soru 0 ile 3 puan arasında değerlendirilecektir. Toplam puan 24'tür.

FORM A – Ön Test

Soru 1 – Yapay zekâ mı, otomasyon mu?

Okulun koridoruna bir hareket sensörü yerleştirilmiştir. Sensör bir öğrencinin geçtiğini fark ettiğinde ışık otomatik olarak yanmaktadır. Sisteme önceden "hareket varsa ışığı aç" komutu verilmiştir.

Sence bu sistem yapay zekâ mıdır? Neden böyle düşünüyorsun?

Yanıt:

Soru 2 – Örneklerden öğrenme ve sınıflandırma

Bir bilgisayara önce birçok "eller yukarıda" ve "eller aşağıda" duruşu gösterilmiştir. Daha sonra bilgisayar daha önce görmediği bir öğrencinin duruşuna bakıp onun "eller yukarıda" grubunda olduğunu söylemiştir.

Bilgisayar yeni öğrencinin hangi grupta olduğunu nasıl tahmin etmiş olabilir? Açıklar mısın?

Yanıt:

Soru 3 – Yapay zekâ gerçekte neyi görüyor?

Bir grup öğrenci geri dönüşüm oyunu hazırlamıştır. Kollar yukarı kaldırıldığında bu hareket "kâğıt", eller yana açıldığında "plastik", çömelme hareketi ise "cam" anlamına gelmektedir. Kamera öğrencinin hareketine bakarak doğru geri dönüşüm kutusunun ışığını yakmaktadır.

Sence bilgisayar gerçekten kâğıdı, plastiği ve camı mı görüyor? Yoksa başka bir şeyi mi görüyor? Açıkla.

Yanıt:

Soru 4 – Farklı kişiler ve veri çeşitliliği

Bir yapay zekâ sistemi yalnızca Ece'nin yaptığı el hareketleri kullanılarak eğitilmiştir. Sistem Ece'nin hareketlerini doğru tanımaktadır. Daha sonra Mehmet aynı hareketleri yaptığında sistem bazen yanlış cevap vermektedir.

Sence bunun nedeni ne olabilir? Sistemin Mehmet'i de daha iyi tanıması için ne yapılabilir?

Yanıt:

Soru 5 – Yapay zekâ hatası ve insan kontrolü

Bir okulda yapay zekâ destekli bir sistem öğrencinin yaptığı hareketi tanıyarak kapıyı açmaktadır. Bir gün öğrenci doğru hareketi yapmasına rağmen sistem onu yanlış tanımış ve kapıyı açmamıştır.

Sence yapay zekâ neden yanlış yapmış olabilir? Böyle bir durumda yalnızca yapay zekânın kararına güvenmek doğru mudur? Neden?

Yanıt:

Soru 6 – Herkes kullanabilir mi?

Bir yardım sistemi, kişi iki kolunu birden yukarı kaldırınca yardım çağırmaktadır. Ancak okulda kollarını kaldırmakta zorlanan bir öğrenci de bulunmaktadır.

Sence bu sistem herkes için uygun mudur? Bu öğrencinin de sistemi kullanabilmesi için nasıl değiştirebilirsin?

Yanıt:

Soru 7 – Çok örnek mi, farklı örnek mi?

İki grup aynı hareketi tanıyan yapay zekâ sistemi hazırlamıştır. Birinci grup aynı öğrencinin aynı hareketini 100 kez göstermiştir. İkinci grup ise 40 örnek kullanmış; fakat örnekleri farklı öğrencilerden, farklı uzaklıklardan ve hareketin biraz farklı biçimlerinden toplamıştır.

Sence hangi sistem yeni bir öğrencinin hareketini daha iyi tanıyabilir? Neden?

Yanıt:

Soru 8 – Yapay zekâ gerçekten gerekli mi?

Bir sınıfta hava karardığında lambaların otomatik olarak yanması istenmektedir. Öğrenciler iki çözüm önermiştir. Birinci çözüm, ışık azaldığında lambayı açan bir ışık sensörü kullanmaktır. İkinci çözüm ise bunun için yapay zekâ sistemi geliştirmektir.

Sen hangi çözümü seçerdin? Bu problemde yapay zekâ kullanmak gerekli midir? Neden?

Yanıt:

FORM B – Son Test

Form B aynı sekiz yeterliği ölçmektedir; ancak öğrencinin Form A'daki senaryoları hatırlayarak yanıt vermesini azaltmak amacıyla bağlamlar değiştirilmiştir.

Soru 1 – Yapay zekâ mı, otomasyon mu?

Bir okul bahçesindeki sulama sistemi her sabah saat 08.00 olduğunda otomatik olarak çalışmaya başlamaktadır. Sisteme daha önce "saat 08.00 ise sulamayı başlat" komutu verilmiştir.

Sence bu sistem yapay zekâ mıdır? Neden?

Yanıt:

Soru 2 – Örneklerden öğrenme ve sınıflandırma

Bir bilgisayara önceden birçok "sağa eğilme" ve "sola eğilme" hareketi gösterilmiştir. Sonra bilgisayar daha önce görmediği bir öğrencinin hareketini "sağa eğilme" olarak belirlemiştir.

Bilgisayar bu yeni hareketin hangi gruba ait olduğuna nasıl karar vermiş olabilir?

Yanıt:

Soru 3 – Sistem gerçekte neyi algılıyor?

Öğrenciler bir trafik oyunu oluşturmuştur. Eller başın üzerindeyse "dur", eller iki yana açılmışsa "geç" anlamı verilmiştir. Kamera hareketi tanıdıktan sonra LEGO trafik ışığı kırmızı veya yeşil yanmaktadır.

Sence yapay zekâ "dur" ve "geç" düşüncelerini mi görüyor, yoksa başka bir şeyi mi tanıyor? Açıklar mısın?

Yanıt:

Soru 4 – Yeni kullanıcılarla karşılaşma

Bir hareket tanıma sistemi yalnızca üç uzun boylu öğrencinin hareketleri kullanılarak eğitilmiştir. Daha sonra daha kısa boylu öğrenciler sistemi denediğinde bazı hareketler yanlış tanınmıştır.

Sence sistem neden hata yapmış olabilir? Daha iyi çalışması için eğitim sırasında neyi değiştirmek gerekir?

Yanıt:

Soru 5 – Hata ve insan denetimi

Bir yapay zekâ sistemi öğrencinin yaptığı yardım hareketini yanlış anlayarak "yardım gerekmiyor" cevabını vermiştir. Öğrenci ise gerçekten yardıma ihtiyaç duymaktadır.

Bu sistem neden hata yapmış olabilir? Böyle önemli bir durumda yapay zekânın kararını bir insanın da kontrol etmesi gerekir mi? Neden?

Yanıt:

Soru 6 – Erişilebilir tasarım

Bir okul oyununda yapay zekânın öğrenciyi tanıması için çocuğun çömelmesi gerekmektedir. Tekerlekli sandalye kullanan bir öğrenci bu hareketi yapamamaktadır.

Sence oyun bu öğrenci için adil ve kullanılabilir midir? Sistemi herkesin kullanabilmesi için nasıl değiştirebilirsin?

Yanıt:

Soru 7 – Veri miktarı ve çeşitliliği

Bir grup yapay zekâ sistemine aynı odada, aynı uzaklıktan ve aynı öğrenciden 80 hareket örneği göstermiştir. Başka bir grup ise 50 örneği farklı öğrencilerden, farklı uzaklıklardan ve biraz farklı açılardan toplamıştır.

Sence daha önce hiç görmediği bir öğrenci geldiğinde hangi sistem daha başarılı olabilir? Neden?

Yanıt:

Soru 8 – AI kullanmak gerekli mi?

Okulun giriş kapısının yalnızca bir düğmeye basıldığında açılması istenmektedir. Bir öğrenci düğmenin kapıyı açmasını sağlayan basit bir sistem önermiş, başka bir öğrenci ise bunun için yapay zekâ kullanmak istemiştir.

Sen hangisini seçerdin? Burada yapay zekâ gerekli midir? Neden?

Yanıt:

Ortak Puanlama Anahtarı

Puan	Değerlendirme ölçütü
0	Yanıt yoktur, soruyla ilgisizdir veya temel kavram tamamen yanlış açıklanmıştır.
1	Öğrenci durumu yüzey özellikleriyle açıklamaktadır. “Robot olduğu için AI”, “kendi yaptığı için AI”, “bilgisayar bildiği için” gibi teknoloji merkezli gerekçeler kullanmaktadır.
2	Temel kavram doğru anlaşılmıştır ancak açıklama eksiktir. Öğrenci örneklerden öğrenme, kural, veri, hata veya kullanıcı farklılığı gibi ilgili kavramlardan en az birini doğru biçimde kullanmakta fakat ilişkileri tam olarak açıklayamamaktadır.
3	Öğrenci mekanizmayı doğru açıklamakta ve yanıtını gerekçelendirmektedir. İlgili soruya göre örneklerden öğrenme ile kuralı, gerçek girdi ile verilen anlamı, veri çeşitliliği ile performansı, AI hatası ile insan kontrolünü veya teknolojinin gerekliliğini açık ve tutarlı biçimde ilişkilendirmektedir.

Her formdaki sekiz görev 0–3 arasında puanlandığında toplam puan 0–24 arasında değişmektedir.

Puanlayıcı Notları: Her Görevin Ölçtüğü Yeterlik

Görev	Form A	Form B
1	Bu soru öğrencinin yalnızca otomatik çalışmayı AI olarak görüp görmediğini ve önceden	Doğru kavramsal açıklamada öğrencinin otomatik çalışmanın tek başına AI anlamına gelmediğini ve sistemin önceden tanımlanmış

Görev	Form A	Form B
	belirlenmiş kural ile öğrenme arasındaki farkı anlayıp anlamadığını ölçmektedir.	bir kuralı uyguladığını belirtmesi beklenmektedir.
2	Burada öğrencinin AI'yı "kendiliğinden bilen" bir sistem olarak mı gördüğü, yoksa önce verilen örneklerden yararlanarak yeni girdiyi sınıflandırdığını mı düşündüğü değerlendirilmektedir.	Bu görev Form A'daki ikinci görevle aynı öğrenme ve sınıflandırma anlayışını farklı bir senaryo üzerinden ölçmektedir.
3	Bu soru öğrencinin sistem tarafından işlenen gerçek girdi ile insanların o girdiye yüklediği anlamı ayırt edip edemediğini değerlendirmektedir.	Üst düzey bir yanıt sistemin vücut duruşunu sınıflandırdığını, "dur" ve "geç" anlamlarının insanlar tarafından kategorilere verildiğini açıklamalıdır.
4	Burada öğrencinin eğitim verilerinin farklı kişileri ve farklı koşulları içermesi gerektiğini fark edip etmediği incelenmektedir.	Bu soru öğrencinin veri çeşitliliğini farklı kullanıcı özellikleri bağlamında genelleyip genelleyemediğini değerlendirmektedir.
5	Bu görev hem model hatasına ilişkin açıklamayı hem de insan kontrolünün gerekliliğini ölçmektedir.	Bu görev model hatasının yanı sıra hatanın sonucunun önemli olduğu durumlarda insan denetimine ilişkin muhakemeyi ölçmektedir.
6	Bu soru erişilebilirlik ve insan merkezli tasarım anlayışını değerlendirmektedir.	Burada öğrencinin yalnızca sorunu fark etmesi değil, alternatif ve daha kapsayıcı bir tasarım önermesi de değerlendirilmektedir.
7	Burada öğrencinin yalnızca veri miktarına mı yoksa veri çeşitliliğine de mi dikkat ettiği değerlendirilmektedir.	Bu görev öğrencinin "daha çok veri her zaman daha iyidir" düşüncesini aşarak veri çeşitliliğinin genelleme açısından önemini açıklayıp açıklayamadığını ölçmektedir.
8	Bu görev öğrencinin AI'yı her problem için tercih edilen teknoloji olarak görüp görmediğini ve teknolojik uygunluk hakkında gerekçe üretip üretemediğini ölçmektedir.	En gelişmiş yanıtın AI'nın kullanılabileceğini söylemek yerine, problemi çözmek için değişken girdileri öğrenmeye veya sınıflandırmaya gerek bulunmadığını ve basit, açık bir kuralın yeterli olduğunu gerekçelendirmesi beklenmektedir.
