# Supplementary material for: When AI Is—and Is Not—Needed: AI Literacy and Computational Thinking in Primary STEM
Source: J Intell. 2026 Oct 12;14(10):257. doi: 10.3390/jintelligence14100257 (PMC13706255; doi:10.3390/jintelligence14100257)
Supplement: Supplementary file 1 [file jintelligence-14-00257-s001.zip › Supplementary_File_S2_Teacher implementation guide_EN_TR.pdf]

Supplementary File S2

Life Studies Teaching Supported by LEGO Education Computer Science & AI

Eight-Week Teacher Implementation Guide

This file provides the written implementation guide that standardized the eight-week intervention reported in the study "When AI Is—and Is Not—Needed: AI Literacy and Computational Thinking in Primary STEM". Part 1 is an English translation; Part 2 reproduces the original Turkish version. Appendix A of the article summarizes the guide (Tables A1–A4).

Part 1. English Translation

1. Purpose of the Guide

This guide was prepared to ensure that the eight-week intervention, in which real-life problems from Life Studies are solved with the LEGO Education Computer Science & AI kit, is conducted according to the same core teaching principles in all experimental groups.

The main goals of the intervention are:

- To develop students' computational thinking skills.
- To help students understand the difference between artificial intelligence and rule-based automation.
- To develop conceptual understanding of training data, classification, data diversity, error, and model limitations through machine learning experiences based on pose recognition.
- To enable students to design AI-based or automation-based solutions to real-life problems.
- To support students in critically evaluating whether AI is necessary for every problem.

2. General Implementation Principles

2.1. Group structure

Each LEGO Education Computer Science & AI kit should be used by four students. Each group has four roles:

1. Design and modeling lead
2. Coding and algorithm lead
3. Data and AI lead
4. Testing and improvement lead

Roles change every week. Over the eight weeks, each student should take on each role twice.

2.2. Role rotation

Sample rotation:

Week	Student A	Student B	Student C	Student D
1	Design	Coding	Data and AI	Testing
2	Testing	Design	Coding	Data and AI
3	Data and AI	Testing	Design	Coding
4	Coding	Data and AI	Testing	Design
5	Design	Coding	Data and AI	Testing
6	Testing	Design	Coding	Data and AI
7	Data and AI	Testing	Design	Coding
8	Coding	Data and AI	Testing	Design

3. The Teacher's Core Role

The teacher:

- Presents the problem.
- Helps students understand the problem.
- Does not give solutions directly.
- Observes the process by moving between the groups.
- Asks neutral questions.
- Asks students to explain their own decisions.
- Lets students discover the distinction between AI and automation instead of stating it directly.
- Does not intervene directly in students' solutions, except in the case of a technical malfunction.

The teacher's core approach should be: **Do not give the answer; ask the question that will make the student think.**

4. Neutral Prompting Questions

When needed, the teacher may use the following questions:

- What exactly is your problem?
- Who is affected by this problem?
- Can you break the problem into smaller parts?
- What should your system do first?
- What should happen next?
- What information does the system use?
- How does the system make a decision?
- Is this decision based on a rule written in advance?
- Does the system learn from new examples?

- What happened on the first try?
- Is there a difference between the result you expected and the actual result?
- Where might the error have come from?
- How can you test this?
- What might happen if another student makes the same movement?
- Would the result change if you used different examples?
- Is the system suitable for everyone?
- Might human control be necessary?
- Is AI really necessary for this problem?
- Could a simpler solution be enough?

5. Prompts the Teacher Should Avoid

The following statements should not be used:

- "This system is not AI."
- "This is just automation."
- "You should collect data from different students."
- "If you use more and more varied data, it will work correctly."
- "AI can always make mistakes."
- "There is no need for AI for this problem."

Such statements make it difficult to observe students' own conceptual development. Instead, neutral questions such as "How does the system work?", "Does it learn from new examples?", and "What happens when a different student makes the same movement?" should be used.

6. The Common Weekly Implementation Cycle

As far as possible, the following cycle should be maintained every week:

1. Present the real-life problem.
2. Have the students discuss the problem.
3. Have the groups choose or narrow down the problem.
4. Assign the roles.
5. Have the students break the problem into sub-parts.
6. Ask them to create an algorithm or a sequence of operations.
7. Let them build the LEGO model.
8. Have them develop the code.
9. In AI weeks, have them train the pose classifier.
10. Ask them to test the system.
11. If there is an error, have them investigate its cause.
12. Give them the opportunity to redesign.

13. Have them complete the group work form.
14. Administer the individual micro-task.
15. Have the students complete the individual participation record.

7. Week 1 — Smart and Safe School

Life Studies context: Me and My School; My Health and Safety

Core problem: Can we design a system that notices a safety problem at school and warns the student?

Technical structure: Events; Simple conditions; Input–process–output; Sensor or event–response relationship

AI use: No. Rule-based automation is developed this week.

Teacher's opening questions

- Which situations at school can be dangerous?
- How could a technological system notice this danger?
- What should the system do when it notices the danger?

Implementation steps

1. Students generate examples of school-safety problems.
2. Each group chooses a problem.
3. The problem is broken into sub-parts.
4. A sequence of operations is created.
5. The LEGO model is built.
6. The system is coded.
7. It is tested in different situations.
8. If there is an error, it is redesigned.

Critical conceptual discussion of the week: Is the system you designed really artificial intelligence?

Neutral questions the teacher may ask

- Does the system learn from new examples?
- Or does it work only according to the rule you gave it?
- Does using a sensor automatically make a system AI?

Direct answer the teacher should not give: "This is not AI." Students are expected to reach this conclusion themselves.

Individual micro-task: Was the system you designed really artificial intelligence? Why?

Data to be collected: Group work form; LEGO product record; Code screenshot; Individual micro-task; Role and participation record; Observation note

8. Week 2 — AI-Supported Movement Coach

Life Studies context: My Health and Safety; Healthy living; Physical activity

Core problem: Can we design an AI system that encourages people to be physically active?

Technical structure: Pose recognition; AI classification; Training examples; Classification; Prediction

AI use: Yes.

Teacher's opening questions

- How could a system understand which movement you are making?
- Is it necessary to teach the movements to the system in advance?
- Could showing one example be enough?

Implementation steps

1. Groups define three or four body poses.
2. A meaning or a LEGO response is assigned to each pose.
3. The AI classifier is trained with examples.
4. Different students perform the poses.
5. The system is tested.
6. Errors are recorded.
7. If necessary, the training examples are changed.

Critical conceptual goal: Students are expected to move toward the understanding that AI does not know the movement by itself; it is first trained with examples.

Neutral questions the teacher may ask

- How many times did you show this pose to the system?
- What might happen if someone else makes the same pose?
- Which examples does the system draw on?
- If it made a wrong prediction, why might that be?

Individual micro-task: How might the AI be recognizing the pose you make?

Data to be collected: Pose classes; Numbers of training examples; First test results; Examples of errors; Micro-task; Group form

9. Week 3 — Recycling Game Through Body Poses

Life Studies context: Nature and Environment; Recycling; Environmental responsibility

Core problem: Can we design an AI-based game that supports making the right choices about recycling?

Technical structure: Pose recognition; Classification; Category matching; Distinguishing input and output

AI use: Yes.

Implementation steps

1. Recycling categories are defined.
2. A pose is chosen for each category.
3. The AI is trained with the poses.
4. A waste item is shown to the student.
5. The student makes the movement that belongs to the correct category.
6. The AI classifies the pose.
7. The LEGO system responds.

Critical accuracy principle: This week the teacher should take particular care to preserve the following distinction: the AI does not recognize the waste; it classifies the body pose made by the student. However, this information should not be given directly at the start of the activity; students should be able to explain the distinction by the end of the task. Critical discussion: What does the system actually recognize?

Neutral questions the teacher may ask

- What does the camera see?
- Which examples did you teach the system?
- Did you show the waste item itself to the system?
- What is the system's input?
- What does the LEGO response depend on?

Individual micro-task: Does this system really recognize the waste, or your body pose? Explain.

Data to be collected: Category–pose matching; Test results; Misclassifications; Group explanation; Micro-task

10. Week 4 — Energy-Saving Smart Classroom

Life Studies context: Nature and Environment; Energy saving; Conscious use of resources

Core problem: Can we design a system that reduces unnecessary energy use in the classroom?

Technical structure: Conditionals; Sensor; If–then structure; Rule-based automation

AI use: No.

Implementation steps

1. The energy problem is identified.
2. Fixed rules are created for the system.
3. An algorithm is prepared with if–then logic.
4. The system is built and coded.
5. It is tested in at least three different situations.
6. The results are recorded.

Critical conceptual goal: Using a sensor does not by itself mean AI.

Neutral questions the teacher may ask

- Which condition does the system check?
- What does it do when the condition is met?
- Who set this rule?
- Does the system learn a new rule by itself?

Individual micro-task: Is every system that uses a sensor artificial intelligence? Explain with an example.

Data to be collected: If–then rules; Test results; Micro-task; Group explanation

11. Week 5 — AI-Supported Safe Pedestrian Crossing

Life Studies context: My Health and Safety; Traffic safety; Needs of different users

Core problem: Can we design an AI system that recognizes different crossing requests from body poses?

Technical structure: Pose recognition; Classification; Different users; Conditionals; Different system outputs

AI use: Yes.

Implementation steps

1. Different crossing requests are defined.
2. A pose is assigned to each request.
3. Examples are collected from different students.
4. The system is trained.
5. The same pose is performed by four different students.
6. The results are compared.

Critical conceptual goal: When the same pose is performed by different people, the AI may not work with the same accuracy.

Neutral questions the teacher may ask

- Does everyone perform the same pose in exactly the same way?
- With whose examples did you train the system?
- Why might the result change when a different student comes?
- How can you diversify the training data?

Individual micro-task: Can the AI recognize everyone who makes the same pose with the same accuracy? Why?

Data to be collected: Tests with different students; Records of correct and incorrect predictions; Student explanations; Micro-task

12. Week 6 — AI for Everyone: An Accessible Assistance System

Life Studies context: My Family and Society; Respect for different needs; Accessibility; Human-centered technology

Core problem: Can we design an AI system that makes daily life easier for people with different needs?

Technical structure: Pose recognition; User needs; AI classification; Accessibility

AI use: Yes.

Implementation steps

1. The group defines a user profile.
2. The problem the user experiences is described.
3. Three different poses and functions are defined.
4. The system is trained and tested.
5. Whether everyone can perform these poses is discussed.
6. Alternative ways of interaction are considered.

Critical conceptual goal: Even if an AI system works technically, it may not be accessible to everyone.

Neutral questions the teacher may ask

- Who is your user?
- Can everyone make this movement?
- How could someone who cannot make it use the system?
- What might happen if the system does not recognize some people?
- What other ways of interaction could be considered?

Individual micro-task: Whom does the system you designed help, and in which situation might it fall short?

Data to be collected: User profile; Purpose of the system; Pose–function matches; Accessibility explanations; Micro-task

13. Week 7 — Why Does AI Make Mistakes?

Life Studies context: Science, Technology and Art; Possibilities and limitations of technology; Critical use of technology

Core problem: Why does AI sometimes recognize the same movement correctly and sometimes incorrectly?

Technical structure: Pose classifier; Training data; Data diversity; Testing; Error; Generalization

AI use: Yes.

Implementation steps

1. Phase 1 — Limited data: The AI system is trained with one or a few students, similar angles, similar distances, and a small number of examples. It is then tested with different students.

2. Phase 2 — Varied data: The model is retrained, this time with different students, different distances, small variations in the pose, and more varied examples. The two models are compared.

Neutral questions the teacher may ask

- Which examples was the first model trained with?
- In which situations did it make mistakes?
- Could many similar examples from the same person be enough?
- What might collecting examples from different people change?
- Why might the second model have worked better or worse?

Statement the teacher should avoid: "If you use more varied data, the model will work better." Students are expected to discover this conclusion themselves.

Individual micro-task: Why did the AI make a mistake, and what kind of training data would it need to work more accurately?

Data to be collected: First-model test results; Second-model test results; Structure of the training data; Explanations of errors; Micro-task

14. Week 8 — Design an AI or Automation Solution for Society

Life Studies context: School; Society; Environment; Health; Safety; Accessibility; Technology

Core problem: Design a solution, using LEGO Education Computer Science & AI, to a real problem encountered at your school, in your neighborhood, or in your daily life. Key decision: The group deliberately chooses one of two options, an AI-based solution or rule-based automation.

AI use: Depends on the group's decision.

Key questions students should answer

1. What is the problem?
2. Who is affected?
3. Is the system AI or automation?
4. If it is AI, what does it recognize?
5. If it is not AI, which rules does it follow?
6. Which inputs does it use?
7. Where might it make mistakes?
8. Is human control necessary?
9. Is AI really necessary?
10. Could a simpler system be enough?

The teacher's role: The teacher does not choose the type of technology and should not say "This problem requires AI" or "Automation would be better here."

Neutral questions the teacher may ask

- Does your system have to learn from new examples?

- Could a fixed rule be enough?
- Does pose recognition really contribute to solving the problem?
- Is a simpler solution possible?

Individual micro-task: Was AI really necessary for the problem you solved? Could a simpler automation system have been enough? Explain your reasoning.

Data to be collected: Final problem definition; Group design form; LEGO product record; Code; Choice of AI or automation; Training data; Test results; Error analysis; Redesign; Final individual micro-task

15. Weekly Time Plan

Suggested standard structure:

Phase	Duration
Presentation and discussion of the problem	10–15 min
Problem selection and decomposition	10–15 min
Algorithm or solution plan	10–15 min
LEGO design and coding	35–45 min
Training and testing	15–20 min
Debugging and redesign	15–20 min
Group form	10 min
Individual micro-task	10 min

The durations are suggested ranges and are adapted to each week's activity (the model is trained only in AI weeks). The total time is about 105–135 min; depending on the school timetable, the activity can be divided into two separate sessions.

16. Implementation Fidelity Checklist

Each week, the teacher or the researcher should complete the following checklist.

Implementation indicator	No	Partly	Yes
The problem was presented to the students in a standard way.	☐	☐	☐
The four roles were used.	☐	☐	☐
The roles were rotated according to the weekly plan.	☐	☐	☐
The students discussed the problem themselves.	☐	☐	☐
The teacher did not give a direct solution.	☐	☐	☐
An algorithm or a sequence of operations was created.	☐	☐	☐
The LEGO model was developed by the students.	☐	☐	☐

Implementation indicator	No	Partly	Yes
The system was tested.	☐	☐	☐
Errors or limitations were discussed.	☐	☐	☐
An opportunity for redesign was given.	☐	☐	☐
In AI weeks, pose recognition was actually used.	☐	☐	☐
Non-AI weeks were not wrongly presented as AI.	☐	☐	☐
The group work form was completed.	☐	☐	☐
The individual micro-task was completed independently.	☐	☐	☐
The teacher did not steer students toward the correct answer.	☐	☐	☐

17. Weekly Researcher Note

At the end of each session, the researcher should write a short note.

Date: ………………………………………………………

Week: ………………………………………………………

Number of participating students: ………………………………………………………

Were there technical problems? ☐ No ☐ Yes — Explanation: ………………………………………………………

Was there any deviation from the planned implementation? ☐ No ☐ Yes — Explanation: ………………………………………………………

Did the students give any notable conceptual explanations? ………………………………………………………

Did anything unexpected happen? ………………………………………………………

Note for the following week: ………………………………………………………

18. Critical Conceptual Boundaries for the Teacher

For the scientific consistency of the study, the following distinctions should be maintained.

Applications that can be treated as AI:

- Pose recognition
- Pose classification
- Building a model with training examples
- Predicting a new pose

Applications that should not be called AI:

- Turning on a light when a sensor detects something
- Starting an operation at a fixed time

- Responding according to an if–then rule
- Repeating a movement with a loop
- Automation that works with fixed rules

One of the strengths of the study is precisely this distinction. Through the process, students should discover that not every system that looks smart is AI.

19. A Final Principle for the Teacher

Throughout the eight-week implementation, the core teaching approach should be as follows: Let the student define the problem. Let the student develop the solution. Let the student notice the error. Let the student discover the difference between AI and automation. Rather than teaching the correct answer, ask the question that will make the student think.

Part 2. Original Turkish Version (Özgün Türkçe Sürüm)

LEGO Computer Science & AI Destekli Hayat Bilgisi Öğretimi

8 Haftalık Öğretmen Uygulama Kılavuzu

1. Kılavuzun Amacı

Bu kılavuz, Hayat Bilgisi temelli gerçek yaşam problemlerinin LEGO Computer Science & AI seti aracılığıyla çözülmesine dayalı 8 haftalık uygulamanın bütün deney gruplarında aynı temel öğretim ilkeleri doğrultusunda yürütülmesini sağlamak amacıyla hazırlanmıştır.

Uygulamanın temel hedefleri şunlardır:

- Öğrencilerin bilgi işlemsel düşünme becerilerini geliştirmek.
- Öğrencilerin yapay zekâ ile kural tabanlı otomasyon arasındaki farkı anlamalarını sağlamak.
- Pozisyon tanımaya dayalı makine öğrenmesi deneyimleri aracılığıyla eğitim verisi, sınıflandırma, veri çeşitliliği, hata ve model sınırlılıkları hakkında kavramsal anlayış geliştirmek.
- Öğrencilerin gerçek yaşam problemlerine yönelik AI veya otomasyon tabanlı çözümler tasarlamalarını sağlamak.
- Öğrencilerin AI’nın her problem için gerekli olup olmadığını eleştirel biçimde değerlendirmelerini desteklemek.

2. Genel Uygulama İlkeleri

2.1. Grup yapısı

Her LEGO Computer Science & AI seti dört öğrenci tarafından kullanılmalıdır. Her grupta dört rol bulunur:

1. Tasarım ve Modelleme Sorumlusu
2. Kodlama ve Algoritma Sorumlusu
3. Veri ve AI Sorumlusu
4. Test ve Geliştirme Sorumlusu

Roller her hafta değiştirilir. Sekiz haftalık süreçte her öğrenci her rolü iki kez üstlenmelidir.

2.2. Rol rotasyonu

Örnek rotasyon:

Hafta	Öğrenci A	Öğrenci B	Öğrenci C	Öğrenci D
1	Tasarım	Kodlama	Veri ve AI	Test
2	Test	Tasarım	Kodlama	Veri ve AI
3	Veri ve AI	Test	Tasarım	Kodlama

Hafta	Öğrenci A	Öğrenci B	Öğrenci C	Öğrenci D
4	Kodlama	Veri ve AI	Test	Tasarım
5	Tasarım	Kodlama	Veri ve AI	Test
6	Test	Tasarım	Kodlama	Veri ve AI
7	Veri ve AI	Test	Tasarım	Kodlama
8	Kodlama	Veri ve AI	Test	Tasarım

3. Öğretmenin Temel Rolü

Öğretmen:

- Problemi sunar.
- Öğrencilerin problemi anlamalarına yardımcı olur.
- Doğrudan çözüm vermez.
- Gruplar arasında dolaşarak süreci gözlemler.
- Nötr sorular sorar.
- Öğrencilerin kendi kararlarını açıklamalarını ister.
- AI ile otomasyon ayrımını doğrudan söylemek yerine öğrencilerin keşfetmesini sağlar.
- Teknik bir arıza dışında öğrencilerin çözümüne doğrudan müdahale etmez.

Öğretmenin temel yaklaşımı şu olmalıdır: **Cevabı verme; öğrencinin düşünmesini sağlayacak soruyu sor.**

4. Kullanılabilecek Nötr Yönlendirme Soruları

Öğretmen ihtiyaç hâlinde şu soruları kullanabilir:

- Probleminiz tam olarak nedir?
- Bu problem kimleri etkiliyor?
- Problemi daha küçük parçalara ayırabilir misiniz?
- Sisteminiz ilk olarak ne yapmalı?
- Bundan sonra ne olmalı?
- Sistem hangi bilgiyi kullanıyor?
- Sistem nasıl karar veriyor?
- Bu karar önceden yazılmış bir kurala mı dayanıyor?
- Sistem yeni örneklerden öğreniyor mu?
- İlk denemede ne oldu?
- Beklediğiniz sonuçla gerçek sonuç arasında fark var mı?
- Hata nereden kaynaklanmış olabilir?
- Bunu nasıl test edebilirsiniz?
- Başka bir öğrenci aynı hareketi yaparsa ne olabilir?

- Daha farklı örnekler kullanırsanız sonuç değişir mi?
- Sistem herkes için uygun mu?
- İnsan kontrolü gerekli olabilir mi?
- Bu problem için gerçekten AI gerekli mi?
- Daha basit bir çözüm yeterli olabilir mi?

5. Öğretmenin Kaçınması Gereken Yönlendirmeler

Aşağıdaki ifadeler kullanılmamalıdır:

- "Bu sistem AI değil."
- "Bu sadece otomasyon."
- "Farklı öğrencilerden veri toplamalısınız."
- "Daha fazla ve çeşitli veri kullanırsanız doğru çalışır."
- "AI her zaman hata yapabilir."
- "Bu problem için AI'ya gerek yok."

Bu tür ifadeler öğrencinin kendi kavramsal gelişimini gözlemlemeyi zorlaştırır. Bunun yerine "Sistem nasıl çalışıyor?", "Yeni örneklerden öğreniyor mu?", "Farklı bir öğrenci aynı hareketi yaptığında ne olur?" gibi nötr sorular kullanılmalıdır.

6. Her Haftanın Ortak Uygulama Döngüsü

Her hafta mümkün olduğunca aşağıdaki döngü korunmalıdır:

1. Gerçek yaşam problemini sun.
2. Öğrencilerin problemi tartışmasını sağla.
3. Grupların problemi seçmesini veya sınırlandırmasını sağla.
4. Rolleri belirle.
5. Problemi alt parçalara ayırmalarını sağla.
6. Algoritma veya işlem sırası oluşturmalarını iste.
7. LEGO modelini kurmalarına izin ver.
8. Kodu geliştirmelerini sağla.
9. AI kullanılan haftalarda pozisyon sınıflandırıcısını eğitmelerini sağla.
10. Sistemi test etmelerini iste.
11. Hata varsa nedenini araştırmalarını sağla.
12. Yeniden tasarım yapmalarına fırsat ver.
13. Grup çalışma formunu tamamlat.
14. Bireysel mikro görevi uygula.
15. Öğrencilerin bireysel katılım kaydını tamamlamasını sağla.

7. 1. Hafta — Akıllı ve Güvenli Okul

Hayat Bilgisi bağlamı: Ben ve Okulum; Sağlığım ve Güvenliğim

Temel problem: Okuldaki bir güvenlik problemini fark eden ve öğrenciyi uyaran bir sistem tasarlayabilir miyiz?

Teknik yapı: Events; Basit koşullar; Girdi–işlem–çıktı; Sensör veya olay–tepki ilişkisi

AI kullanımı: Hayır. Bu hafta kural tabanlı otomasyon geliştirilir.

Öğretmenin açılış soruları

- Okulda hangi durumlar tehlikeli olabilir?
- Bir teknolojik sistem bu tehlikeyi nasıl fark edebilir?
- Sistem tehlikeyi fark ettiğinde ne yapmalıdır?

Uygulama adımları

1. Öğrenciler okul güvenliğiyle ilgili problem örnekleri üretir.
2. Her grup bir problem seçer.
3. Problem alt parçalara ayrılır.
4. İşlem sırası oluşturulur.
5. LEGO modeli kurulur.
6. Sistem kodlanır.
7. Farklı durumlarda test edilir.
8. Hata varsa yeniden tasarlanır.

Haftanın kritik kavramsal tartışması: Tasarladığınız sistem gerçekten yapay zekâ mı?

Öğretmenin sorabileceği nötr sorular

- Sistem yeni örneklerden öğreniyor mu?
- Yoksa sadece sizin verdiğiniz kurala göre mi çalışıyor?
- Sensör kullanmak bir sistemi otomatik olarak AI yapar mı?

Öğretmenin vermemesi gereken doğrudan cevap: "Bu AI değildir." Öğrencinin bu sonuca kendisinin ulaşması beklenmelidir.

Bireysel mikro görev: Tasarladığınız sistem gerçekten yapay zekâ mıydı? Neden?

Toplanacak veriler: Grup çalışma formu; LEGO ürün kaydı; Kod ekran görüntüsü; Bireysel mikro görev; Rol ve katılım kaydı; Gözlem notu

8. 2. Hafta — AI Destekli Hareket Koçu

Hayat Bilgisi bağlamı: Sağlığım ve Güvenliğim; Sağlıklı yaşam; Fiziksel hareket

Temel problem: İnsanların hareket etmelerini destekleyen bir AI sistemi tasarlayabilir miyiz?

Teknik yapı: Pozisyon tanıma; AI sınıflandırması; Eğitim örnekleri; Sınıflandırma; Tahmin

AI kullanımı: Evet.

Öğretmenin açılış soruları

- Bir sistem sizin hangi hareketi yaptığınızı nasıl anlayabilir?
- Sisteme hareketleri önceden öğretmek gerekir mi?
- Bir örnek göstermek yeterli olabilir mi?

Uygulama adımları

1. Gruplar üç veya dört beden pozisyonu belirler.
2. Her pozisyona bir anlam veya LEGO tepkisi atanır.
3. AI sınıflandırıcı örneklerle eğitilir.
4. Farklı öğrenciler pozisyonları yapar.
5. Sistem test edilir.
6. Hatalar kaydedilir.
7. Gerekiyorsa eğitim örnekleri değiştirilir.

Kritik kavramsal amaç: Öğrencinin şu anlayışa yaklaşması beklenir: AI hareketi kendiliğinden bilmiyor; önce örneklerle eğitiliyor.

Öğretmenin sorabileceği nötr sorular

- Sisteme bu pozisyonu kaç kez gösterdiniz?
- Aynı pozisyonu başka biri yaparsa ne olabilir?
- Sistem hangi örneklerden yararlanıyor?
- Yanlış tahmin yaptıysa neden olabilir?

Bireysel mikro görev: Yapay zekâ yaptığın pozisyonu nasıl tanıyor olabilir?

Toplanacak veriler: Pozisyon sınıfları; Eğitim örneği sayıları; İlk test sonuçları; Hata örnekleri; Mikro görev; Grup formu

9. 3. Hafta — Beden Hareketleriyle Geri Dönüşüm Oyunu

Hayat Bilgisi bağlamı: Doğa ve Çevre; Geri dönüşüm; Çevre sorumluluğu

Temel problem: Geri dönüşüm konusunda doğru seçim yapmayı destekleyen AI tabanlı bir oyun tasarlayabilir miyiz?

Teknik yapı: Pozisyon tanıma; Sınıflandırma; Kategori eşleştirme; Girdi ve çıktı ayrımı

AI kullanımı: Evet.

Uygulama adımları

1. Geri dönüşüm kategorileri belirlenir.
2. Her kategori için bir pozisyon seçilir.
3. AI pozisyonlarla eğitilir.
4. Öğrenciye atık örneği gösterilir.
5. Öğrenci doğru kategoriye ait hareketi yapar.
6. AI pozisyonu sınıflandırır.
7. LEGO sistemi tepki verir.

Kritik doğruluk ilkesi: Bu hafta öğretmen şu ayrımı özellikle korumalıdır: AI atığı tanımamaktadır. AI öğrencinin yaptığı beden pozisyonunu sınıflandırmaktadır. Ancak bu bilgi etkinlik başında doğrudan verilmemeli; öğrenciler görev sonunda bu ayrımı açıklayabilmelidir. Kritik tartışma: Sistem gerçekte neyi tanıyor?

Öğretmenin sorabileceği nötr sorular

- Kamera ne görüyor?
- Sisteme hangi örnekleri öğrettiniz?
- Atığın kendisini sisteme gösterdiniz mi?
- Sistemin girdisi nedir?
- LEGO tepkisi neye göre oluşuyor?

Bireysel mikro görev: Bu sistem gerçekte atığı mı, yoksa beden pozisyonunu mu tanıyor? Açıkla.

Toplanacak veriler: Kategori–pozisyon eşleştirmesi; Test sonuçları; Yanlış sınıflandırmalar; Grup açıklaması; Mikro görev

10. 4. Hafta — Enerji Tasarruflu Akıllı Sınıf

Hayat Bilgisi bağlamı: Doğa ve Çevre; Enerji tasarrufu; Kaynakların bilinçli kullanımı

Temel problem: Sınıfta gereksiz enerji kullanımını azaltan bir sistem tasarlayabilir miyiz?

Teknik yapı: Conditionals; Sensör; Eğer–ise yapısı; Kural tabanlı otomasyon

AI kullanımı: Hayır.

Uygulama adımları

1. Enerji problemi belirlenir.
2. Sistem için sabit kurallar oluşturulur.
3. Eğer–ise mantığıyla algoritma hazırlanır.
4. Sistem kurulur ve kodlanır.
5. En az üç farklı durumda test edilir.
6. Sonuçlar kaydedilir.

Kritik kavramsal amaç: Sensör kullanmak tek başına AI anlamına gelmez.

Öğretmenin sorabileceği nötr sorular

- Sistem hangi koşulu kontrol ediyor?
- Şart gerçekleştiğinde ne yapıyor?
- Bu kuralı kim belirledi?
- Sistem kendi kendine yeni bir kural öğreniyor mu?

Bireysel mikro görev: Sensör kullanan her sistem yapay zekâ mıdır? Bir örnekle açıkla.

Toplanacak veriler: Eğer–ise kuralları; Test sonuçları; Mikro görev; Grup açıklaması

11. 5. Hafta — AI Destekli Güvenli Yaya Geçidi

Hayat Bilgisi bağlamı: Sağlığım ve Güvenliğim; Trafik güvenliği; Farklı kullanıcı ihtiyaçları

Temel problem: Farklı geçiş taleplerini beden pozisyonlarından tanıyan bir AI sistemi tasarlayabilir miyiz?

Teknik yapı: Pozisyon tanıma; Sınıflandırma; Farklı kullanıcılar; Conditionals; Farklı sistem çıktıları

AI kullanımı: Evet.

Uygulama adımları

1. Farklı geçiş talepleri belirlenir.
2. Her talebe bir pozisyon atanır.
3. Farklı öğrencilerden örnekler toplanır.
4. Sistem eğitilir.
5. Aynı pozisyon dört farklı öğrenci tarafından yapılır.
6. Sonuçlar karşılaştırılır.

Kritik kavramsal amaç: Aynı pozisyon farklı kişiler tarafından yapıldığında AI aynı doğrulukta çalışmayabilir.

Öğretmenin sorabileceği nötr sorular

- Aynı pozisyon herkes tarafından tam olarak aynı mı yapılır?
- Sistemi kimlerin örnekleriyle eğittiniz?
- Farklı bir öğrenci geldiğinde sonuç neden değişebilir?
- Eğitim verisini nasıl çeşitlendirebilirsiniz?

Bireysel mikro görev: AI aynı pozisyonu yapan bütün insanları aynı doğrulukta tanıyabilir mi? Neden?

Toplanacak veriler: Farklı öğrenci testleri; Doğru–yanlış tahmin kayıtları; Öğrenci açıklamaları; Mikro görev

12. 6. Hafta — Herkes İçin AI: Erişilebilir Yardım Sistemi

Hayat Bilgisi bağlamı: Ailem ve Toplum; Farklı ihtiyaçlara saygı; Erişilebilirlik; İnsan merkezli teknoloji

Temel problem: Farklı ihtiyaçları olan insanların günlük yaşamını kolaylaştıran bir AI sistemi tasarlayabilir miyiz?

Teknik yapı: Pozisyon tanıma; Kullanıcı ihtiyacı; AI sınıflandırması; Erişilebilirlik

AI kullanımı: Evet.

Uygulama adımları

1. Grup bir kullanıcı profili belirler.
2. Kullanıcının yaşadığı problem tanımlanır.
3. Üç farklı pozisyon ve işlev belirlenir.

4. Sistem eğitilir ve test edilir.
5. Herkesin bu pozisyonları yapıp yapamayacağı tartışılır.
6. Alternatif etkileşim yolları düşünülür.

Kritik kavramsal amaç: AI sistemi teknik olarak çalışsa bile herkes için erişilebilir olmayabilir.

Öğretmenin sorabileceği nötr sorular

- Kullanıcınız kim?
- Bu hareketi herkes yapabilir mi?
- Yapamayan biri sistemi nasıl kullanabilir?
- Sistem bazı kişileri tanımazsa ne olabilir?
- Başka hangi etkileşim yolları düşünülebilir?

Bireysel mikro görev: Tasarladığınız sistem kime yardım ediyor ve hangi durumda yetersiz kalabilir?

Toplanacak veriler: Kullanıcı profili; Sistem amacı; Pozisyon–işlev eşleştirmeleri; Erişilebilirlik açıklamaları; Mikro görev

13. 7. Hafta — Yapay Zekâ Neden Hata Yapar?

Hayat Bilgisi bağlamı: Bilim, Teknoloji ve Sanat; Teknolojinin olanakları ve sınırlılıkları; Eleştirel teknoloji kullanımı

Temel problem: AI neden aynı hareketi bazen doğru, bazen yanlış tanıyor?

Teknik yapı: Pozisyon sınıflandırıcısı; Eğitim verisi; Veri çeşitliliği; Test; Hata; Genelleme

AI kullanımı: Evet.

Uygulama adımları

1. Aşama 1 — Sınırlı veri: AI sistemi tek veya az sayıda öğrenci, benzer açı, benzer uzaklık ve az sayıda örnek ile eğitilir. Ardından farklı öğrencilerle test edilir.
2. Aşama 2 — Çeşitli veri: Model yeniden eğitilir. Bu kez farklı öğrenciler, farklı uzaklıklar, küçük pozisyon değişiklikleri ve daha çeşitli örnekler kullanılır. İki model karşılaştırılır.

Öğretmenin sorabileceği nötr sorular

- İlk model hangi örneklerle eğitildi?
- Hangi durumda hata yaptı?
- Aynı kişiden çok sayıda benzer örnek yeterli olabilir mi?
- Farklı kişilerden örnek toplamak neyi değiştirebilir?
- İkinci model neden daha iyi veya daha kötü çalışmış olabilir?

Öğretmenin kaçınması gereken ifade: "Daha çeşitli veri kullanırsanız model daha iyi çalışır." Bu sonucu öğrencinin kendisinin keşfetmesi beklenmelidir.

Bireysel mikro görev: AI neden hata yaptı ve daha doğru çalışması için nasıl bir eğitim verisi kullanmak gerekir?

Toplanacak veriler: İlk model test sonuçları; İkinci model test sonuçları; Eğitim verisi yapısı; Hata açıklamaları; Mikro görev

14. 8. Hafta — Toplum İçin AI veya Otomasyon Çözümü Tasarla

Hayat Bilgisi bağlamı: Okul; Toplum; Çevre; Sağlık; Güvenlik; Erişilebilirlik; Teknoloji

Temel problem: Okulunuzda, mahallenizde veya günlük yaşamınızda karşılaşılan gerçek bir probleme LEGO Computer Science & AI kullanarak çözüm tasarlayın. Temel karar: Grup şu iki seçenekten birini bilinçli olarak seçer: AI tabanlı çözüm veya kural tabanlı otomasyon.

AI kullanımı: Grup kararına bağlı.

Öğrencilerin cevaplaması gereken temel sorular

1. Problem nedir?
2. Kimleri etkiliyor?
3. Sistem AI mı, otomasyon mu?
4. AI ise neyi tanıyor?
5. AI değilse hangi kurallarla çalışıyor?
6. Hangi girdileri kullanıyor?
7. Nerede hata yapabilir?
8. İnsan kontrolü gerekli mi?
9. AI gerçekten gerekli mi?
10. Daha basit bir sistem yeterli olabilir mi?

Öğretmenin rolü: Öğretmen teknoloji türünü seçmez. "Bu problem AI gerektiriyor." veya "Burada otomasyon daha iyi olur." dememelidir.

Öğretmenin sorabileceği nötr sorular

- Sisteminiz yeni örneklerden öğrenmek zorunda mı?
- Sabit bir kural yeterli olabilir mi?
- Pozisyon tanıma gerçekten probleme katkı sağlıyor mu?
- Daha basit bir çözüm mümkün mü?

Bireysel mikro görev: Çözdüğünüz problem için gerçekten AI gerekli miydi? Daha basit bir otomasyon sistemi yeterli olabilir miydi? Gerekçenle açıkla.

Toplanacak veriler: Final problem tanımı; Grup tasarım formu; LEGO ürün kaydı; Kod; AI veya otomasyon seçimi; Eğitim verisi; Test sonuçları; Hata analizi; Yeniden tasarım; Final bireysel mikro görev

15. Haftalık Süre Planı

Önerilen standart yapı:

Aşama	Süre
Problemin sunulması ve tartışma	10–15 dakika
Problem seçimi ve ayrıştırma	10–15 dakika
Algoritma/çözüm planı	10–15 dakika
LEGO tasarım ve kodlama	35–45 dakika
Eğitim ve test	15–20 dakika
Hata ayıklama ve yeniden tasarım	15–20 dakika
Grup formu	10 dakika
Bireysel mikro görev	10 dakika

Süreler önerilen aralıklardır ve haftanın etkinliğine göre uyarlanır (modelin eğitilmesi yalnızca yapay zekâ haftalarında yer alır). Toplam süre yaklaşık 105–135 dakikadır; uygulama okulun ders programına göre iki ayrı oturuma bölünebilir.

16. Uygulama Sadakati Kontrol Listesi

Her hafta öğretmen veya araştırmacı şu kontrol listesini tamamlamalıdır.

Uygulama göstergesi	Hayır	Kısmen	Evet
Problem öğrencilere standart biçimde sunuldu.	☐	☐	☐
Dört rol uygulandı.	☐	☐	☐
Roller haftalık plana göre döndürüldü.	☐	☐	☐
Öğrenciler problemi kendileri tartıştı.	☐	☐	☐
Öğretmen doğrudan çözüm vermedi.	☐	☐	☐
Algoritma veya işlem sırası oluşturuldu.	☐	☐	☐
LEGO modeli öğrenciler tarafından geliştirildi.	☐	☐	☐
Sistem test edildi.	☐	☐	☐
Hata veya sınırlılık tartışıldı.	☐	☐	☐
Yeniden tasarım fırsatı verildi.	☐	☐	☐
AI kullanılan haftalarda pozisyon tanıma gerçekten kullanıldı.	☐	☐	☐
AI olmayan haftalar yanlış biçimde AI olarak sunulmadı.	☐	☐	☐
Grup çalışma formu tamamlandı.	☐	☐	☐
Bireysel mikro görev bağımsız olarak tamamlandı.	☐	☐	☐
Öğretmen doğru cevabı yönlendirmedi.	☐	☐	☐

17. Haftalık Araştırmacı Notu

Her uygulama sonunda araştırmacı kısa bir not yazmalıdır.

Tarih: ……………………………………………………

Hafta: ……………………………………………………

Katılan öğrenci sayısı: ……………………………………………………

Teknik sorun yaşandı mı? ☐ Hayır ☐ Evet — Açıklama: ……………………………………………………

Planlanan uygulamadan sapma oldu mu? ☐ Hayır ☐ Evet — Açıklama: ……………………………………………………

Öğrencilerin dikkat çeken bir kavramsal açıklaması oldu mu? ……………………………………………………

Beklenmeyen bir durum yaşandı mı? ……………………………………………………

Sonraki haftaya ilişkin not: ……………………………………………………

18. Öğretmen İçin Kritik Kavramsal Sınırlar

Araştırmanın bilimsel tutarlılığı açısından şu ayrımlar korunmalıdır.

AI olarak ele alınabilecek uygulamalar:

- Pozisyon tanıma
- Pozisyon sınıflandırma
- Eğitim örnekleriyle model oluşturma
- Yeni bir pozisyonun tahmin edilmesi

AI olarak adlandırılmaması gereken uygulamalar:

- Sensör gördüğünde ışık yakma
- Sabit bir saatte işlem başlatma
- Eğer–ise kuralına göre tepki verme
- Döngüyle hareket tekrarlama
- Sabit kurallarla çalışan otomasyon

Araştırmanın güçlü noktalarından biri tam olarak bu ayrımdır. Öğrenciler süreç içinde şunu keşfetmelidir: Her akıllı görünen sistem AI değildir.

19. Öğretmen İçin Son İlke

Sekiz haftalık uygulama boyunca temel öğretim yaklaşımı şu olmalıdır: Problemi öğrencinin tanımlamasına izin ver. Çözümü öğrencinin geliştirmesine izin ver. Hatayı öğrencinin fark etmesine izin ver. AI ile otomasyon arasındaki farkı öğrencinin keşfetmesine izin ver. Doğru cevabı öğretmek yerine, öğrencinin düşünmesini sağlayacak soruyu sor.
